# Identification of the Protein Glycation Sites in Human Myoglobin as Rapidly Induced by d-Ribose

**DOI:** 10.3390/molecules26195829

**Published:** 2021-09-26

**Authors:** Jing-Jing Liu, Yong You, Shu-Qin Gao, Shuai Tang, Lei Chen, Ge-Bo Wen, Ying-Wu Lin

**Affiliations:** 1 School of Chemistry and Chemical Engineering, University of South China, Hengyang 421001, China; 2006000753@usc.edu.cn (J.-J.L.); 20212005110245@usc.edu.cn (S.T.); 20192005110153@usc.edu.cn (L.C.); 2 Laboratory of Protein Structure and Function, University of South China, Hengyang 421001, China; 2006001977@usc.edu.cn (Y.Y.); 2014001853@usc.edu.cn (S.-Q.G.); wengebo@usc.edu.cn (G.-B.W.)

**Keywords:** diabetes, protein glycation, human myoglobin, d-ribose, glycosylation sites

## Abstract

Protein glycation is an important protein post-translational modification and is one of the main pathogenesis of diabetic angiopathy. Other than glycated hemoglobin, the protein glycation of other globins such as myoglobin (Mb) is less studied. The protein glycation of human Mb with ribose has not been reported, and the glycation sites in the Mb remain unknown. This article reports that d-ribose undergoes rapid protein glycation of human myoglobin (HMb) at lysine residues (K34, K87, K56, and K147) on the protein surface, as identified by ultra-high performance liquid chromatography-mass spectrometry (UHPLC-MS) and electrospray ionization tandem mass spectrometry (ESI-MS/MS). Moreover, glycation by d-ribose at these sites slightly decreased the rate of the met heme (Fe^III^) in reaction with H_2_O_2_ to form a ferryl heme (Fe^IV^=O). This study provides valuable insight into the protein glycation by d-ribose and provides a foundation for studying the structure and function of glycated heme proteins.

## 1. Introduction

Diabetes is one of the most common metabolic diseases, and recently its prevalence has been on the rise. According to the global estimates of diabetes prevalence for 2013, the number of patients with diabetes worldwide is expected to increase to 592 million by 2035 [1]. A persistently high level of blood carbohydrates may cause serious damage to the human body by accelerating the process of protein glycation [2]. In the primary stage, the aldehyde group of sugars tends to react with α- and ε-amino groups (*N*-terminal and Arg/Lys residues), forming a Schiff base, which undergoes a rearrangement and yields more stable derivatives called Amadori compounds [3]. As an important protein post-translational modification (PTM) [4], protein glycation may cause loss of function and is one of the main pathogenesis of diabetic angiopathy [5]. In addition to glucose, the amount of ribose and fructose was shown to have significantly increased in diabetic blood and tissues, which induced the glycation of proteins such as α-synuclein, serum, and other cellular proteins [6,7,8,9].

For the investigation of protein glycations, the most in-depth study is on glycated hemoglobin (Hb) [10,11,12,13,14,15,16,17]. In 2010–2011, the American Diabetes Association (ADA) and the World Health Organization (WHO) recommended HbA1c, an adduct of glucose and Hb, as a new standard for the diagnosis of diabetes [18]. Meanwhile, different carbohydrates and proteins in the human body may undergo protein glycation, leading to alterations of the protein structure and function [6,7,8,10,11,12,13,14,15,16,17]. For example, glucose and ribose were found to undergo protein glycation with the Hb [10,11,12,13,14,15,16,17]. The glycation sites of glucose in the Hb were identified previously, including in the major sites of β-V1, α-K16, and β-K66, and other Lys residues in both *α* and *β* subunits, whereas the structures of the glycation products were not reported [10,12]. In general, both aldoses and ketoses may react with the protein to yield Amadori and Heyns compounds, respectively [19]. Moreover, Siddiqui et al. showed that the ribose-induced glycation of Hb has resulted in perturbations of both the secondary and tertiary structure [15,16]. Instead, acetyl salicylic acid (ASA, Aspirin) and *p*-nitro-benzoic acid (NBA) can prevent the glycation of the Hb with a mechanism of acetylation of the Lys residues [17]. 

**Figure 1:**
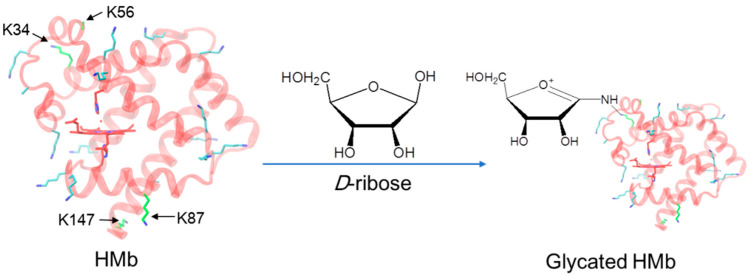
The X-ray crystal structure of the K45R/C110A HMb mutant (**left**, PDB code 3RGK) [30]. The heme active site and the location of the Lys residues are shown for clarification. Note that the conformation of the side chain of K56 was too flexible to be determined. The protein glycation of the HMb by d-ribose is indicated by the arrow (**right**).

Comparatively, the protein glycation of other globins in the organism, such as myoglobin ((Mb), Figure 1, left), is less studied [20,21,22,23,24]. In addition to the biological function of O_2_ storage and transportation, Mb may also exhibit multiple biological functions, such as serving as a nitric oxide (NO) scavenger and a hypoxic nitrite reductase (NIR) [25,26,27], as well as a peroxidase using H_2_O_2_ as an oxidant [28,29]. Mb mainly exists in the cytoplasm of cardiomyocytes and in skeletal muscle cells. When these cells are damaged, Mb can quickly enter the blood circulation and undergo protein glycation with carbohydrates such as glucose, resulting in modifications to the protein structure and function [20]. Recently, Grune et al. showed that the proteasome prefers to degrade the glucose-modified Mb [23].

As a naturally occurring pentose sugar, d-ribose is widely used in food, medicine, cosmetics, etc. [31]. A certain amount of the d-ribose introduced to cardiomyocytes may cause unexpected side effects, such as the protein glycation of the d-ribose with Mb. It has been shown that ribose causes internal cross-links of the horse heart Mb by glycation [21]. Meanwhile, the protein glycation of ribose and the human Mb (termed HMb) has not been reported, and the glycation sites in the Mb remain unknown. Motivated by the progress, we were interested in studying the protein glycation of HMb by d-ribose (Figure 1, right). As demonstrated in what follows, we found that d-ribose rapidly induces the glycation of the HMb and further identified four glycation sites by both UHPLC-MS and ESI-MS/MS spectrometric studies.

## 2. Results and Discussion

### 2.1. UV-Vis Studies

The HMb was expressed in *E. Coli.* BL21(DE3) cells and purified using a protocol as previously reported [29]. To test the protein glycation of the HMb, we performed reactions with the d-ribose or d-glucose at 37 °C, pH 7.0, for 1–24 h. The UV-Vis spectra of the reaction solution were recorded at 1, 4, 8, 12, and 24 h, respectively. As shown in Figure S1A, the HMb exhibited the UV-Vis spectrum with characteristic absorption (Soret band, 409 nm; visible bands, ~500 and 630 nm), similar to that reported previously in pH 6.0 (409.5, 504, and 633 nm) [32]. Upon reaction with d-ribose or d-glucose, although the sugar did not cause blue- or red-shifts in the Soret band within 24 h, the d-ribose caused a large decrease in the intensity compared to that of the d-glucose (Figure S1B). In a previous study, we showed by SDS-PAGE that no degradation of the protein was observed after incubating with the d-ribose for two days [33]. It was also shown that the incubation of Mb with d-glucose under the same conditions for six days resulted in a decrease of the Soret band [22]. These observations suggest that the incubation with d-ribose altered the protein structure of the HMb, likely by rapid protein glycation.

### 2.2. Mass Spectrometry Analysis

#### 2.2.1. Direct Flow Injection

**Figure 2:**
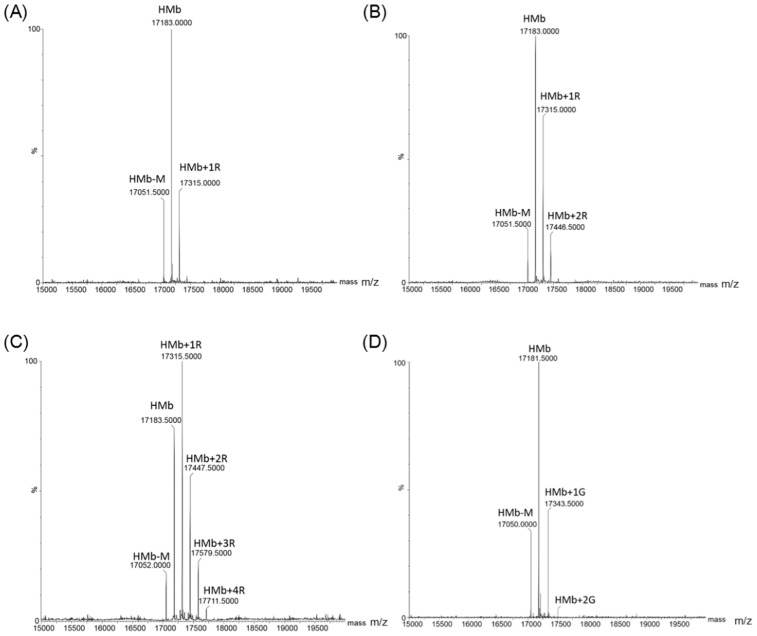
ESI-MS spectra of the HMb after reacting with d-ribose for 1 h (**A**), 2 h (**B**), and 4 h (**C**), respectively. The spectrum of HMb after reacting with d-glucose for 24 h (**D**) was shown for comparison.

To confirm the occurrence of protein glycation of the HMb in the reaction, we performed mass spectrometric studies to detect the ribosylated HMb and the glycosylated human Mb. Once purified, the HMb exhibited an ESI-MS spectrum with a major peak of 17,183 Da, which corresponds to the mass of apo-protein. The mass of 17,052 Da was due to the absence of a Met residue (149 Da) at the *N*-terminus (termed HMb-M, Figure S2). In the reaction with d-ribose for 1, 2, and 4 h (Figure 2A–C and Figure S3A–C), the mass spectra showed an increased number of peaks, which correspond to the HMb covalently attached to one, two, and four d-ribose molecules, respectively, with each attachment increasing the mass by 132 Da (150 Da of ribose minus 18 Da of H_2_O). The relative errors of mass determination relative to theoretical values were shown in Table S1. As listed in Table 1, the long-time incubations at 37 °C produced more ribosylated products. For example, the attachment of even seven d-ribose molecules was observed for 24 h. By contrast, in the reaction with d-glucose, only the attachment of one or two d-glucose molecules to the HMb was observed after 24 h (Figure 2D and Figure S3D, and Table 1). Note that there were other minor peaks, which could hardly be identified as possible modification products. These observations indicate that compared with d-glucose, d-ribose undergoes rapid protein glycation with HMb at multiple sites.

#### 2.2.2. UHPLC-MS Studies

**Figure 3:**
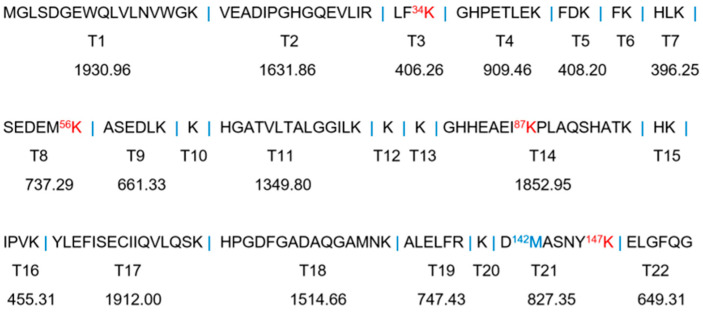
The amino acid sequence of HMb and the expected peptide fragments (T1–T22) by trypsin digestion. The molecular weight of each peptide fragment and the four identified Lys residues are labeled.

In an early study, Shapiro et al. showed that the glycation sites of human Hb by glucose involved several Lys residues [10]. To identify the glycosylation sites of HMb induced by d-ribose, we performed trypsin digestion studies of the ribosylated HMb and determined the mass spectra of the protein fragments (Figure 3). This method was based on the supposition that the glycated Lys and Arg residues may resist the cleavage by the trypsin. As a result, the peptides modified by the d-ribose to the Lys/Arg will generate new signals in both the extracted ion chromatogram (EIC) and the mass spectra. 

**Figure 4:**
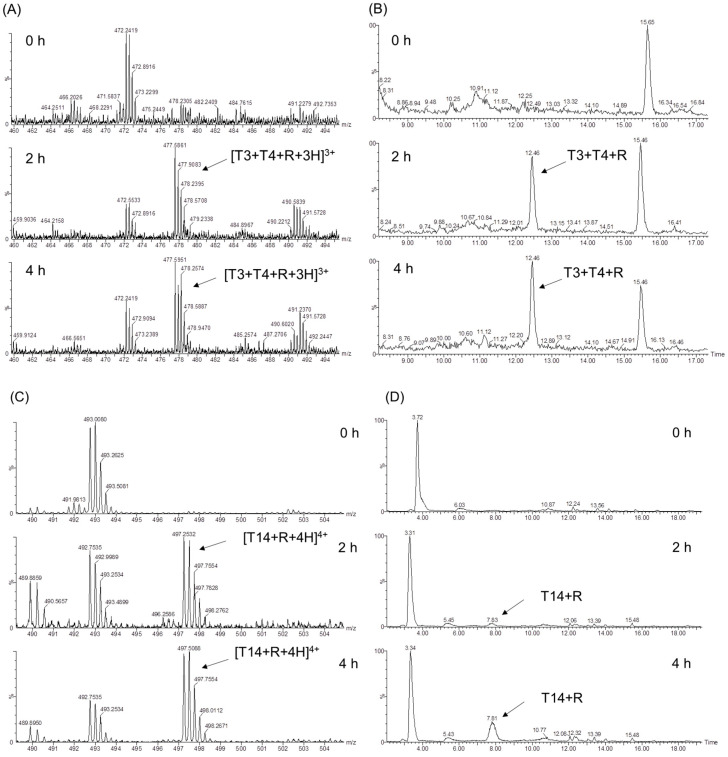
ESI-MS spectra (**A**,**C**) and extracted ion chromatograms (EICs) (**B**,**D**) of the trypsin digestion products of HMb induced by d-ribose at different times (0, 2, and 4 h).

As expected, the UHPLC-MS fragmentation of the incubated HMb/d-ribose revealed newly generated peaks from the EIC (Figure 4 and Figure S4). As shown in Figure 4A, the signal of *m*/*z* 447 appeared at the incubation time for 2 and 4 h, corresponding to the new peak at 12.46 min in the EIC diagram (Figure 4B). According to the trypsin digestion specificity, K34 is one of the digestion sites, producing the peptides T3 and T4 (Figure 3). Meanwhile, when the ribose-induced glycosylation occurred at this site, the peptides of T3 and T4 may not be cleaved with the modification of K34. Therefore, the newly generated triply charged mass signal (*m*/*z* 447) can be considered the glycosylated peptide of T3–T4, with the covalent attachment of one ribose molecule, termed [T3 + T4 + R + 3H]^3+^. Hence, K34 was presumably one of the sites where the d-ribose and the HMb underwent protein glycation. 

In the case of peptide T14, K87 is adjacent to P88 and is thus hard to be cleaved by the trypsin at this site. As shown in Figure 4C,D, the glycosylation of K87 likely occurred after incubating for 2–4 h, generating a quadruply charged mass signal of *m*/*z* = 497, which can be assigned to [T14 + R + 4H]^4+^. Moreover, the glycosylation at other Lys sites was observed after incubating for 4 h. As shown in Figure S3, the quadruply charged mass signal of *m*/*z* 380 and *m*/*z* 804 could be assigned to [T8 + T9 + R + 4H]^4+^ and [T21 + T22 + O + R + 2H]^2+^, as a result of the glycosylation at K56 and K147, respectively. Note that the M142 in peptide T21 was likely oxidized to the sulfoxide form (SO-Met) during the incubation, as observed for the other heme proteins such as neuroglobin and cytochrome *c* [34,35]. 

According to the relative intensity of mass signals at different reaction times, the K34 was presumably glycosylated by d-ribose first, followed by K56, K87, and K147, in that order. This observation might be attributed to the location of these Lys residues on the protein surface, as well as their local micro-environments (Figure 1, left). It should be noted that within the incubation for 4 h, no Arg residue was found to undergo the glycation reaction, which suggests that the side chain of the Lys is more prone to protein glycation with the d-ribose than that of the Arg. These observations agree with those Lys sites on the protein surface identified for glucose-induced glycation of Hb, and no Arg site was identified [10,12]. 

#### 2.2.3. Analysis of Tandem Mass Spectra

**Figure 5:**
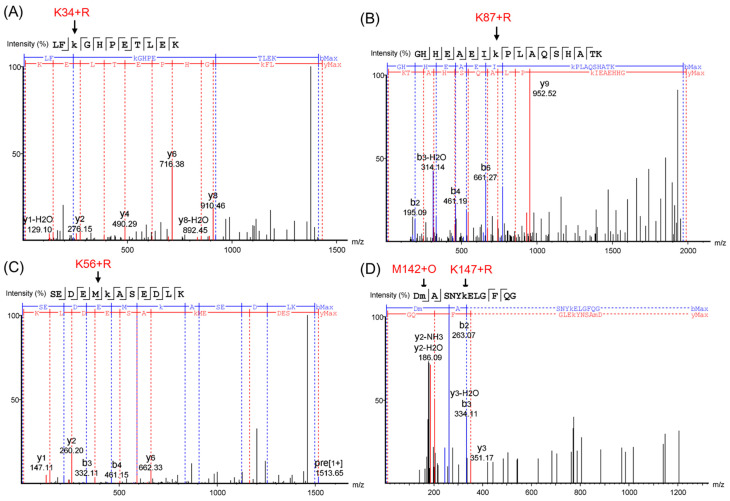
ESI-MS/MS spectra of peptide fragments: The triply protonated tryptic glycosylated HMb peptide LFK(+132.04)GHPETLEK at *m*/*z* 477.58 (**A**); the triply protonated tryptic glycosylated HMb peptide GHHEAEIK(+132.04)PLAQSHATK at *m*/*z* 662.67 (**B**); the doubly protonated tryptic glycosylated HMb peptide SEDEMK(+132.04)ASEDLK at *m*/*z* 757.33 (**C**); and the triply protonated tryptic glycosylated HMb peptide DM(+15.99)ASNYK(+132.04)ELGFQG at *m*/*z* 804.35 (**D**), respectively. The glycation sites of the Lys and the oxidation of the Met142 are indicated by arrows.

To further confirm the glycation sites in the HMb, we performed ESI-MS/MS studies and analyzed the spectra by PEAKS Studio X+ (Bioinformatics Solutions Inc., Waterloo, ON, Canada). The PEAKS DB was set up to search the uniport_homo sapiens database (version 201907, entries 20428), assuming trypsin as the digestion enzyme. As shown in Figure 5, for analysis of the peptide fragments, the results (Table S2 in details) further verified the four glycation sites of K34, K56, K87, and K147, and the oxidation of Met142. Accordingly, to the signals with a loss of 18 u, the ribose-derived Amodori compound formed oxonium ions by the loss of one water molecule, as proposed previously for glycated peptides and proteins [19,36].

### 2.3. Stopped-Flow Kinetic Studies

**Figure 6:**
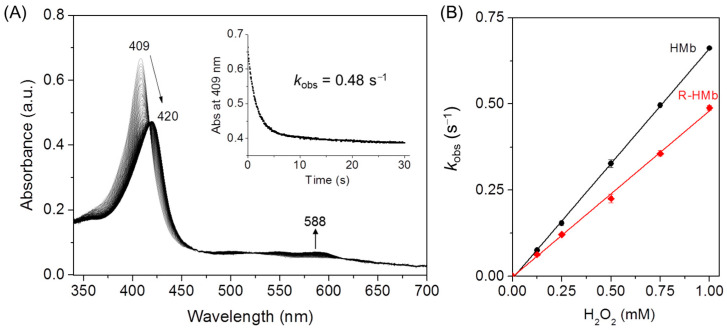
(**A**) The stopped-flow spectra of the ribosylated HMb (R-HMb) in reaction with H_2_O_2_ (1 mM) at pH 7.0 for 30 s. Inset: The decay of the Soret band at 409 nm. (**B**) The plots of the observed rate constants versus the H_2_O_2_ concentrations, with the results of the HMb shown for comparison.

It is important to elucidate the structure–function relationship of the heme proteins with structural alterations by PTMs [4,37]. To evaluate the effect of ribose glycation on HMb’s function, we performed kinetic studies by reaction of the protein with H_2_O_2_. As shown in Figure 6A, upon mixing with H_2_O_2_, the met ribosylated HMb rapidly converted from met (Fe^III^) to ferryl form (Fe^IV^=O, 420 and 588 nm), similar to those observed in previous studies [22,29]. By fitting the decay of the Sort band to the single-exponential function, the obtained *k*_obs_ values for the formation of ferryl heme were linearly dependent on the concentration of H_2_O_2_ (Figure 6B). The linear fit yielded the apparent rate constant (*k*_1_) of 580 (mol/L)^−1^ s^−1^ for the ribosylated HMb, which is ~13% lower than that of the HMb determined under the same conditions (670 (mol/L)^−1^ s^−1^). Note that the *k*_1_ value of the HMb in this study is slightly higher than previously reported by Hirota et al. (510 (mol/L)^−1^ s^−1^) [29], likely due to the slightly different reaction conditions. The slight decrease in the rate of H_2_O_2_ activation for the ribosylated HMb suggests that the glycation slightly altered the heme active site structure, although the modified Lys sites are remote from the heme center. In-depth studies on the structure and function of the ribosylated HMb are currently ongoing in our lab.

## 3. Conclusions

This study has provided evidence to show that d-ribose rapidly induces the protein glycation of HMb, which occurs at four Lys residues (K34, K56, K87 and K147) after incubating with d-ribose for 4 h. As identified by UHPLC-MS and ESI-MS/MS spectrometric studies, K34 was the first site to be modified with d-ribose, followed by K56, K87, and K147, while no glycation of Arg was observed. Moreover, the glycation by d-ribose at these sites slightly decreased the rate of the heme iron in reaction with H_2_O_2_. Therefore, this study provides valuable insight into the protein glycation by d-ribose and provides a foundation for studying the structure and function of glycated heme proteins. 

## 4. Materials and Methods

### 4.1. Protein Preparation

The pET3a plasmid DNA containing the gene of wild-type (WT) human myoglobin was a gift from Prof. Shun Hirota, Nara Institute of Science and Technology (NAIST), Japan. The protein was expressed in BL21(DE3) and purified using a procedure described in the literature [29]. The purity of the HMb was confirmed by the mass spectrum and the ratio between the absorbance at 280 and 409 nm (Abs_409nm_/Abs_280nm_ > 5.0). 

### 4.2. In Vitro Protein Glycation 

The d-ribose or d-glucose with the final concentration of 0.5 mmol/L was added into the HMb solution (0.1 mmol/L, final concentration) [15,20,23]. After the filtration and sterilization, the mixed solution was incubated at 37 °C for 1–24 h. Under the same conditions, the HMb solution without the added d-ribose or d-glucose was used as the control.

### 4.3. UV-Vis Studies

The UV-Vis spectra of the HMb were recorded in 20 mmol/L potassium phosphate buffer (pH 7.0) on Agilent 8453 diode array spectrometer (Agilent Technologies, Inc., Santa Clara, CA, USA). The protein concentration was determined with an extinction coefficient of ε_409_ = 153 L/mmol·cm [32]. The UV-Vis spectra of the HMb incubation with d-ribose or d-glucose were recorded before and after reacting for 1, 4, 8, 12, and 24 h, respectively.

### 4.4. Mass Spectrometry

#### 4.4.1. Flow Injection Analysis (FIA)

A protein mass spectrum measurement was carried out on a G2-XS QToF mass spectrometer (Waters, Milford, MA, USA). The desalted protein solution (~20 μmol/L) was mixed with 1% (*v*/*v*) formic acid and distilled water in a volume ratio of 1:1:8, and transferred into the mass spectrometric source for measurement under the positive mode in the direct flow injection mode. ESI experiments were carried out under the following constant instrumental conditions: Capillary voltage: 3.5 kV; Sample cone: 50 V; Extraction cone: 4 V; Source temperature: 120 °C; Desolvation temperature: 400 °C; Cone gas: 50 L/h; Desolvation gas: 600 L/h; Injection volume: 50 μL; and Flow rate: 10 μL/min. The multiple *m*/*z* peaks were transformed to the protein molecular weight by using the MaxEnt1 software. 

#### 4.4.2. UHPLC-MS Studies

For the analysis of the protein fragments, the glycated HMb samples induced by d-ribose for 0, 2, and 4 h were digested by trypsin. After being denatured in a metal bath with dithiothreitol (10 mmol/L, final concentration) in the ammonium bicarbonate buffer (50 mmol/L, pH 8.5) at 60 °C for 1 h, the HMb/d-ribose incubation samples (~100 μg of protein) were cooled to room temperature and then added to iodoacetamide (10 mmol/L, final concentration) to react in the dark for 0.5 h. A proteomics grade trypsin (at 1:30 wt. ratio) was added to the reconstituted protein, and the solution was incubated at 37 °C for 15 h to ensure complete trypsin digestion, and then filtered by a 10 KD centrifuge tube to remove trypsin. The filtrate was freeze-dried for the analysis of mass spectrometry.

An Ultra-high Performance Liquid Chromatography (UHPLC-MS) analysis was performed using an ACUQITY UPLC coupled online to a G2-XS QToF mass spectrometer (Waters) with an electrospray injection. The samples were separated using a 2.1 × 50 mm C18 reverse-phase column (Acquity UPLC BEH, Waters). The elution was performed by using 0.1% (*v*/*v*) HCOOH in distilled water (eluent A) and 1% (*v*/*v*) HCOOH in acetonitrile (eluent B), with a flow rate of 0.2 mL min^−1^; the elution started with 5% (*v*/*v*) solvent B for 5 min, followed by a linear gradient from 5 to 35 % (*v*/*v*) B in 45 min. The MS/MS spectra were obtained by collision-induced dissociation (CID) with a ramp collision energy of 20–30 V.

#### 4.4.3. Analysis of Tandem Mass Spectra

The tandem mass spectra were processed by PEAKS Studio version X+ (Bioinformatics Solutions Inc., Waterloo, ON, Canada). The PEAKS DB was set up to search the uniport_homo sapiens database (version201907, entries 20428), assuming trypsin as the digestion enzyme. The PEAKS DB was used for searching with a fragment ion mass tolerance of 0.02 Da and a parent ion tolerance of 7 ppm. Carbamidomethylation (+57.02 Da) was specified as the fixed modification. Oxidation (Met, +15.99 Da), deamidation (NQ, +0.98 Da), acetylation (Protein *N*-terminus, +42.01 Da), and ribose glycosylation (Lys and Arg, +132.04 Da) were specified as the variable modifications. The peptides with −10lgP ≥ 20 and the proteins with −10lgP ≥ 20 and containing at least 1unique peptide were filtered. 

### 4.5. Stopped-Flow Kinetic Studies

The kinetic UV-Vis studies of the glycated HMb induced by d-ribose for 24 h with H_2_O_2_ were carried out at 25 °C on a dual mixing stopped-flow spectrophotometer (SF-61DX2 Hi-Tech KinetAsyst^TM^) (Hi-Tech Scientific, Bradford-on-Avon, UK). Typically, one syringe contained 7~10 μM protein (100 mmol/L potassium phosphate buffer, pH 7.0), and the second syringe contained H_2_O_2_, with concentrations ranging from 0.25 mmol/L to 2.0 mmol/L, as determined with ε_240nm_ = 39.4 L/mmol·cm [38]. The reaction was initiated by mixing an equal volume of solutions from both syringes. A total of 50 time-dependent spectra were collected over 10 s from 350 to 700 nm at 25 °C. The decay of the Soret band at 409 nm obeyed pseudo-first-order kinetics, and the curve was fitted to a single-exponential function to calculate the *k*_obs_ values. The HMb was used for control studies under the same conditions.

## Figures and Tables

**Table 1:** The protein glycation products of HMb as induced by d-ribose or d-glucose at different reaction times.

Time (h)	Ribosylated HMb	Glycosylated HMb
1	HMb + 1R	/
2	HMb + 1R, HMb + 2R	/
4	HMb + 1R, HMb + 2R, HMb + 3R, HMb + 4R	/
8	HMb + 1R, HMb + 2R, HMb + 3R, HMb + 4R	/
12	HMb + 1R, HMb + 2R, HMb + 3R, HMb + 4R, HMb + 5R	/
24	HMb + 2R, HMb + 3R, HMb + 4R, HMb + 5R, HMb + 6R, HMb + 7R	HMb + 1G, HMb + 2G

Note: “HMb”, “R”, and “G” are the abbreviations for human myoglobin, d-ribose, and d-glucose, respectively, and “/” indicates “not detectable”.

## Data Availability

The datasets for this manuscript can be obtained from the corresponding author upon reasonable request.

## Supplementary Materials

The following are available online. Figure S1: The UV-Vis spectra of HMb incubated with d-ribose and d-glucose; Figure S2: The ESI-MS spectrum of the expressed and purified HMb; Figure S3: The original multiply charged series in the ESI-MS spectra of the HMb after reacting with the d-ribose at different hours; Figure S4: The ESI-MS spectra and the extracted ion chromatograms of trypsin digestion products of the HMb reacted with the d-ribose for 0, 2 or 4 h; Table S1: The relative errors in mass determination relative to the theoretical values as analyzed in Figure 2; and Table S2: The results of the peptide MS/MS analysis with PEAKS.

## Sample Availability

The samples of the compounds and/or corresponding spectra are available from the authors.

## References

[1] Guariguata L., Whiting D.R., Hambleton I., Beagley J., Linnenkamp U., Shaw J.E. (2014). Global estimates of diabetes prevalence for 2013 and projections for 2035. Diabetes Res. Clin. Pract..

[2] Giardino I., Edelstein D., Brownlee M. (1994). Nonenzymatic glycosylation in vitro and in bovine endothelial cells alters basic fibroblast growth factor activity. A model for intracellular glycosylation in diabetes. J. Clin. Investig..

[3] Schalkwijk C.G., Ligtvoet N., Twaalfhoven H., Jager A., Blaauwgeers H.G., Schlingemann R.O., Tarnow L., Parving H.H., Stehouwer C.D., Van Hinsbergh V.W. (1999). Amadori albumin in type 1 diabetic patients: Correlation with markers of endothelial function, association with diabetic nephropathy, and localization in retinal capillaries. Diabetes.

[4] Lin Y.-W. (2018). Structure and function of heme proteins regulated by diverse post-translational modifications. Arch. Biochem. Biophys..

[5] Adrover M., Mariño L., Sanchis P., Pauwels K., Kraan Y., Lebrun P., Vilanova B., Muñoz F., Broersen K., Donoso J. (2014). Mechanistic Insights in Glycation-Induced Protein Aggregation. Biomacromolecules.

[6] Chen Y., Yu L., Wang Y., Wei Y., Xu Y., He T., He R. (2019). d-Ribose contributes to the glycation of serum protein. Biochim. Biophys. Acta (BBA)-Mol. Basis Dis..

[7] Carulli S., Calvano C.D., Palmisano F., Pischetsrieder M. (2011). MALDI-TOF MS Characterization of Glycation Products of Whey Proteins in a Glucose/Galactose Model System and Lactose-free Milk. J. Agric. Food Chem..

[8] Schalkwijk C.G., Stehouwer C.D.A., Van Hinsbergh V.W.M. (2004). Fructose-mediated non-enzymatic glycation: Sweet coupling or bad modification. Diabetes/Metab. Res. Rev..

[9] Li S., Wang J., Xiao Y., Zhang L., Fang J., Yang N., Zhang Z., Nasser M.I., Qin H. (2021). D-ribose: Potential clinical applications in congestive heart failure and diabetes, and its complications (Review). Exp. Ther. Med..

[10] Shapiro R., McManus M., Zalut C., Bunn H. (1980). Sites of nonenzymatic glycosylation of human hemoglobin A. J. Biol. Chem..

[11] Bakhti M., Habibi-Rezaei M., Moosavi-Movahedi A., Khazaei M. (2007). Consequential Alterations in Haemoglobin Structure upon Glycation with Fructose: Prevention by Acetylsalicylic Acid. J. Biochem..

[12] Wang S.-H., Wang T.-F., Wu C.-H., Chen S.-H. (2014). In-Depth Comparative Characterization of Hemoglobin Glycation in Normal and Diabetic Bloods by LC-MSMS. J. Am. Soc. Mass Spectrom..

[13] Van Steen S.C., Schrieks I.C., Hoekstra J.B., Lincoff A.M., Tardif J.-C., Mellbin L.G., Rydén L.E., Grobbee D., DeVries J.H., on behalf of the AleCardio Study Group (2017). The haemoglobin glycation index as predictor of diabetes-related complications in the AleCardio trial. Eur. J. Prev. Cardiol..

[14] Chen X., Su T., Chen Y., He Y., Liu Y., Xu Y., Wei Y., Li J., He R. (2017). d-Ribose as a Contributor to Glycated Haemoglobin. EBioMedicine.

[15] Siddiqui Z., Ishtikhar M., Moinuddin, Ahmad S. (2018). d-Ribose induced glycoxidative insult to hemoglobin protein: An approach to spot its structural perturbations. Int. J. Biol. Macromol..

[16] Siddiqui Z., Faisal M., Alatar A.R., Ahmad S. (2019). Prevalence of auto-antibodies against D-ribose-glycated-hemoglobin in diabetes mellitus. Glycobiology.

[17] Ghazanfari-Sarabi S., Habibi-Rezaei M., Eshraghi-Naeeni R., Moosavi-Movahedi A.A. (2019). Prevention of haemoglobin glycation by acetylsalicylic acid (ASA): A new view on old mechanism. PLoS ONE.

[18] Little R.R., Rohlfing C.L. (2013). The long and winding road to optimal HbA1c measurement. Clin. Chim. Acta.

[19] Frolov A., Hoffmann P., Hoffmann R. (2006). Fragmentation behavior of glycated peptides derived from D-glucose, D-fructose and D-ribose in tandem mass spectrometry. J. Mass Spectrom..

[20] Roy A., Sil R., Chakraborti A.S. (2009). Non-enzymatic glycation induces structural modifications of myoglobin. Mol. Cell. Biochem..

[21] Bokiej M., Livermore A.T., Harris A.W., Onishi A.C., Sandwick R.K. (2011). Ribose sugars generate internal glycation cross-links in horse heart myoglobin. Biochem. Biophys. Res. Commun..

[22] You Y., Liu F., Du K.-J., Wen G.-B., Lin Y.-W. (2014). Structural and functional alterations of myoglobin by glucose-protein interactions. J. Mol. Model..

[23] Raupbach J., Ott C., König J., Grune T. (2020). Proteasomal degradation of glycated proteins depends on substrate unfolding: Preferred degradation of moderately modified myoglobin. Free. Radic. Biol. Med..

[24] Zhang P., Yuan H., Xu J., Wang X.-J., Gao S.-Q., Tan X., Lin Y.-W. (2020). A Catalytic Binding Site Together with a Distal Tyr in Myoglobin Affords Catalytic Efficiencies Similar to Natural Peroxidases. ACS Catal..

[25] Gladwin M.T., Kim-Shapiro D.B. (2008). The functional nitrite reductase activity of the heme-globins. Blood.

[26] Kamga C., Krishnamurthy S., Shiva S. (2012). Myoglobin and mitochondria: A relationship bound by oxygen and nitric oxide. Nitric Oxide.

[27] Wu L.-B., Yuan H., Gao S.-Q., You Y., Nie C.-M., Wen G.-B., Lin Y.-W., Tan X. (2016). Regulating the nitrite reductase activity of myoglobin by redesigning the heme active center. Nitric Oxide.

[28] Witting P.K., Mauk A.G., Lay P. (2002). Role of Tyrosine-103 in Myoglobin Peroxidase Activity: Kinetic and Steady-State Studies on the Reaction of Wild-Type and Variant Recombinant Human Myoglobins with H_2_O_2_. Biochemestry.

[29] Nagao S., Asami O., Yasui H., Hirota S. (2011). Efficient reduction of Cys110 thiyl radical by glutathione in human myoglobin. Biochim. Biophys. Acta (BBA) Proteins Proteom..

[30] Hubbard S.R., Hendrickson W.A., Lambright D.G., Boxer S.G. (1990). X-ray crystal structure of a recombinant human myoglobin mutant at 2·8 Å resolution. J. Mol. Biol..

[31] Park Y.-C., Choi J.-H., Bennett G.N., Seo J.-H. (2006). Characterization of d-ribose biosynthesis in Bacillus subtilis JY200 deficient in transketolase gene. J. Biotechnol..

[32] Ikeda-Saito M., Hori H., Andersson L.A., Prince R.C., Pickering I.J., George G.N., Sanders C.R., Lutz R.S., McKelvey E.J., Mattera R. (1992). Coordination structure of the ferric heme iron in engineered distal histidine myoglobin mutants. J. Biol. Chem..

[33] You Y., Liu F., Gao S.-Q., Lin Y.-W., Wen G.-B. (2016). D-ribose induced rapid non-enzymatic glycation of human myoglobin. Med. Sci. J. Cent. South China.

[34] Wang Z., Ando Y., Nugraheni A.D., Ren C., Nagao S., Hirota S. (2014). Self-oxidation of cytochrome c at methionine80 with molecular oxygen induced by cleavage of the Met–heme iron bond. Mol. BioSyst..

[35] Liu H.-X., Li L., He B., Gao S.-Q., Wen G.-B., Lin Y.-W. (2018). Neuroglobin is capable of self-oxidation of methionine64 introduced at the heme axial position. Dalton Trans..

[36] Soboleva A., Schmidt R., Vikhnina M., Grishina T., Frolov A. (2017). Maillard Proteomics: Opening New Pages. Int. J. Mol. Sci..

[37] Liu C., Yuan H., Liao F., Wei C.-W., Du K.-J., Gao S.-Q., Tan X., Lin Y.-W. (2019). Unique Tyr-heme double cross-links in F43Y/T67R myoglobin: An artificial enzyme with a peroxidase activity comparable to that of native peroxidases. Chem. Commun..

[38] Nelson D.P., Kiesow L.A. (1972). Enthalpy of decomposition of hydrogen peroxide by catalase at 25 °C (with molar extinction coefficients of H_2_O_2_ solutions in the UV). Anal. Biochem..

