# Supplementary material for: Identification of the Protein Glycation Sites in Human Myoglobin as Rapidly Induced by d-Ribose
Source: Molecules. 2021 Sep 26;26(19):5829. doi: 10.3390/molecules26195829 (PMC8512392; doi:10.3390/molecules26195829)
Supplement: Supplementary file 1 [file molecules-26-05829-s001.zip › molecules-1368237-supplementary.pdf]

Supporting Information

Identification of the Protein Glycation Sites in Human Myoglobin as Rapidly Induced by *D*-Ribose

Jing-Jing Liu,[a] Yong You,[b] Shu-Qin Gao,[b] Shuai Tang,[a] Lei Chen,[a] Ge-Bo Wen,[b] and Ying-Wu Lin*[a, b]

[a] *School of Chemistry and Chemical Engineering, University of South China, Hengyang 421001, China;*

[b] *Laboratory of Protein Structure and Function, University of South China, Hengyang 421001, China.*

** Correspondence: ywlin@usc.edu.cn*

Contents

1. Figure S1. The UV-Vis spectra of HMb incubated with D-ribose and D-glucose. P. S2

2. Figure S2. The ESI-MS spectrum of the expressed and purified HMb. P. S2

3. Figure S3. The original multiply-charged series in the ESI-MS spectra of HMb after reacting with *D*-ribose at different hours. P. S3

4. Figure S4. The ESI-MS spectra and the extracted ion chromatograms (EICs) of the trypsin digestion products of the Mb induced by *D*-ribose for 0, 2 or 4 h. P. S4

5. Table S1. The relative errors in mass determination relative to the theoretical values as analyzed in Figure 2. P. S5

6. Table S2. The results of the peptide MS/MS analysis with PEAKS. P. S6

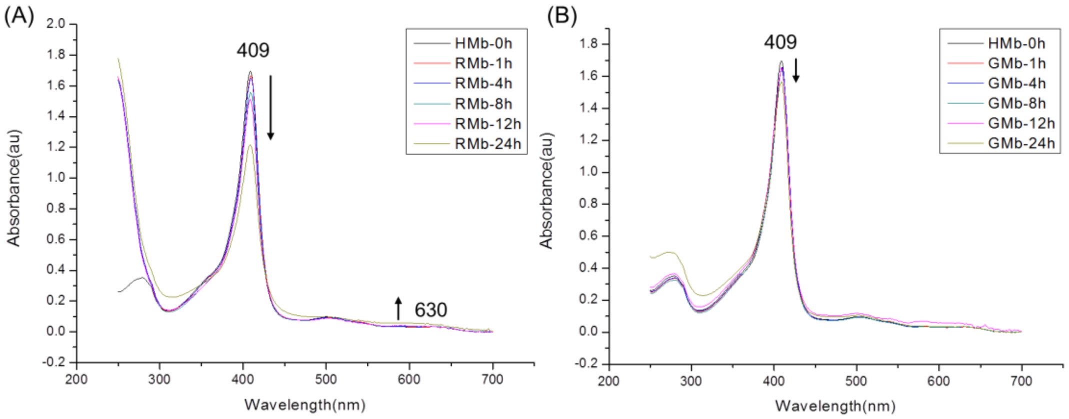

Figure S1. The UV-Vis spectra of HMb incubated with D-ribose (A) or D-glucose (B) (0.5 mM) at pH 7.0, 37 ℃ for 0-24 h.

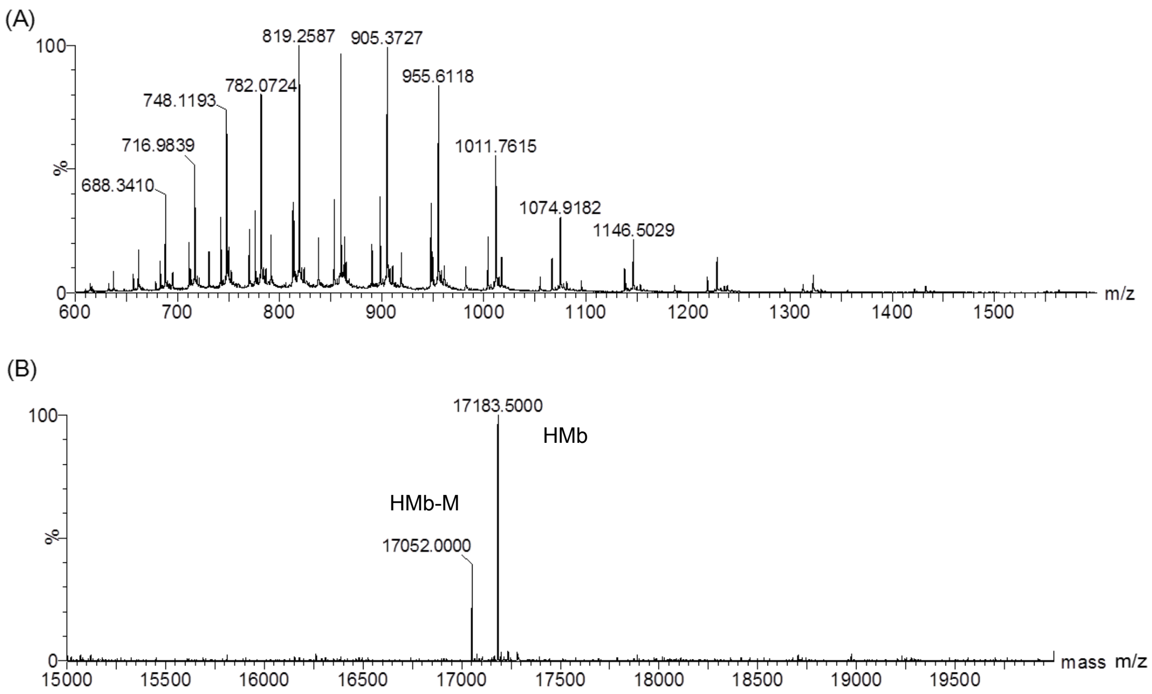

Figure S2. The ESI-MS spectrum of the expressed and purified HMb: (A) Original multiply-charged series. (B) The MaxEnt survey spectrum showing the major component. Calculated, 17184 Da; Observed, 17183.5±0.5 Da. The mass of 17052 Da is attributed to the absence of a Met residue (149 Da) at the N-terminus (termed HMb-M).

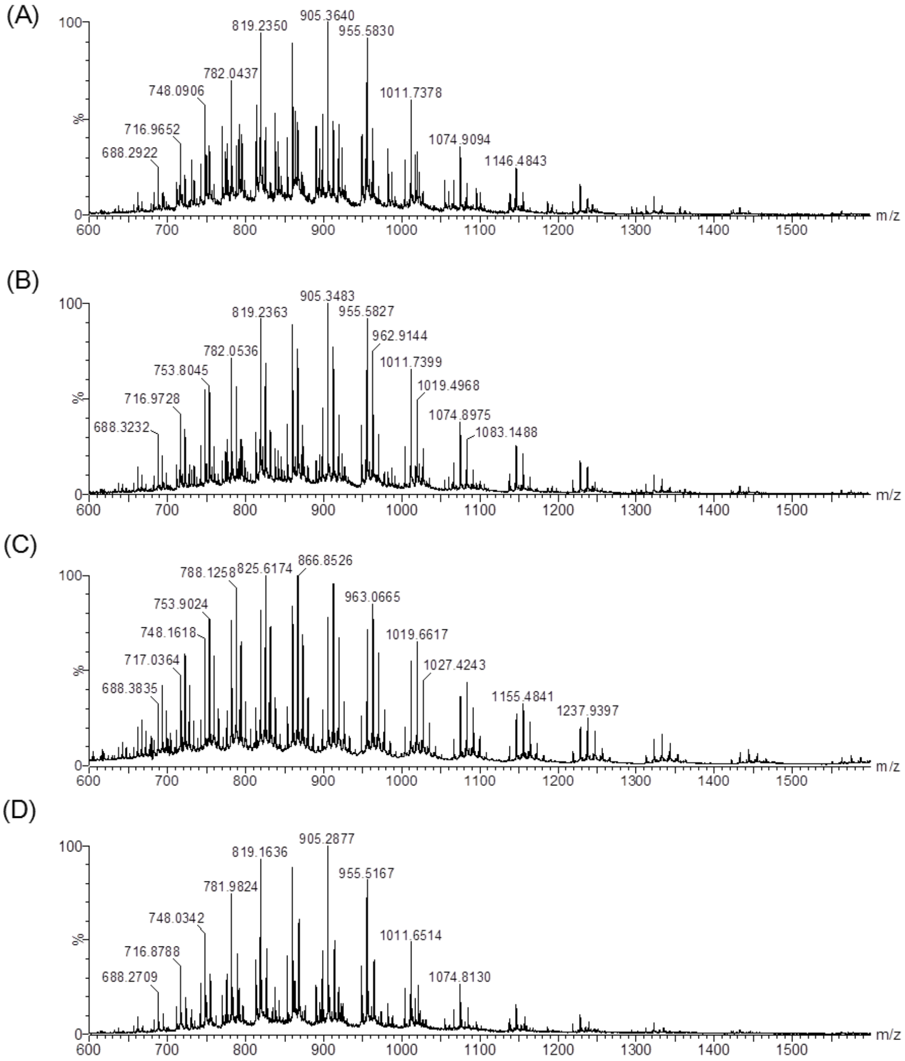

Figure S3. The original multiply-charged series in ESI-MS spectra of HMb after reacting with *D*-ribose for 1 h (A), 2 h (B), and 4 h (C), respectively. The spectrum of HMb after reacting with *D*-glucose for 24 h (D) was shown for comparison.

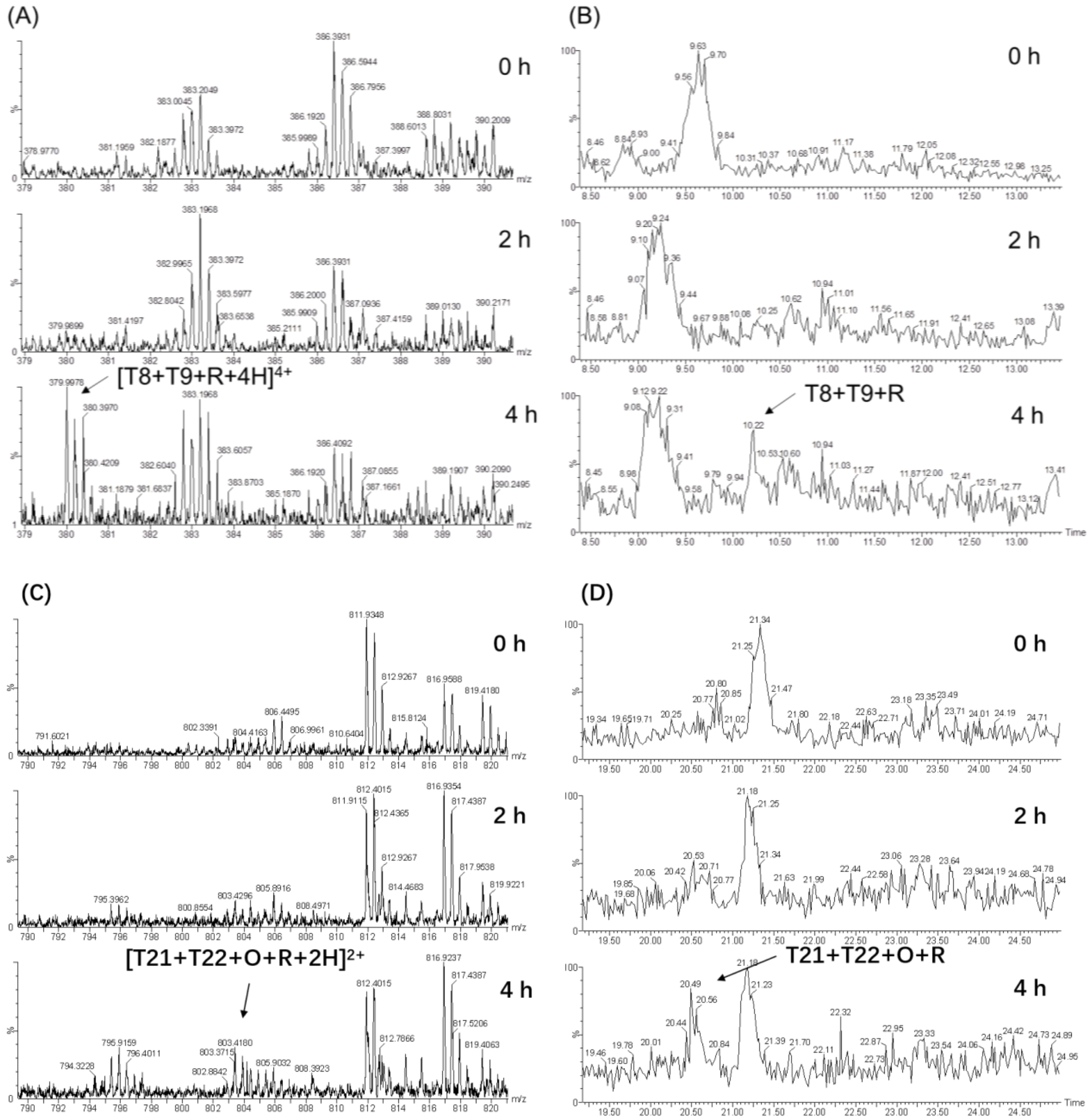

Figure S4. The ESI-MS spectra (A, C) and the extracted ion chromatograms (EICs) (B, D) of the trypsin digestion products of the HMb induced by *D*-ribose for 0, 2 and 4 h.

Table S1. The relative errors in mass determination relative to the theoretical values as analyzed in Figure 2.

Time (hours)	Protein	Ion m/z	Theoretical m/z	m/z error (ppm)
1	HMb	17183.0	17184	-58
	HMb + 1R	17315.0	17316	-58
2	HMb	17183.0	17184	-58
	HMb + 1R	17315.0	17316	-58
	HMb + 2R	17446.5	17448	-86
4	HMb	17183.5	17184	-29
	HMb + 1R	17315.5	17316	-29
	HMb + 2R	17447.5	17448	-29
	HMb + 3R	17579.5	17580	-28
	HMb + 4R	17711.5	17712	-28
24	HMb	17181.5	17184	-145
	HMb + 1G	17343.5	17346	-144

Table S2. The results of the peptide MS/MS analysis with PEAKS.

Peptide	mass	m/z	z	protein	ion	ion m/z	theoretical m/z	m/z error
LFK(+132.04)GHPET LEK	1429.7429	715.8788	2	P02144	b2-H2O	243.1342	243.1497	0.0156
LFK(+132.04)GHPET LEK	1429.7429	715.8788	2	P02144	b7-H2O	923.4473	923.4604	0.0131
LFK(+132.04)GHPET LEK	1429.7429	715.8788	2	P02144	y1	147.1124	147.1128	0.0004
LFK(+132.04)GHPET LEK	1429.7429	715.8788	2	P02144	y2	276.1548	276.1554	0.0005
LFK(+132.04)GHPET LEK	1429.7429	715.8788	2	P02144	y3	389.2369	389.2394	0.0025
LFK(+132.04)GHPET LEK	1429.7429	715.8788	2	P02144	y4	490.2859	490.2871	0.0013
LFK(+132.04)GHPET LEK	1429.7429	715.8788	2	P02144	y5	619.3273	619.3297	0.0024
LFK(+132.04)GHPET LEK	1429.7429	715.8788	2	P02144	y6	716.3804	716.3824	0.002
LFK(+132.04)GHPET LEK	1429.7429	715.8788	2	P02144	y7	853.4365	853.4413	0.0049
LFK(+132.04)GHPET LEK	1429.7429	715.8788	2	P02144	y8	910.4608	910.4628	0.002
LFK(+132.04)GHPET LEK	1429.7429	715.8788	2	P02144	y1-H2O	129.1019	129.1022	0.0004
LFK(+132.04)GHPET LEK	1429.7429	715.8788	2	P02144	y2-H2O	258.1442	258.1448	0.0006
LFK(+132.04)GHPET LEK	1429.7429	715.8788	2	P02144	y6-H2O	698.3727	698.3719	-0.0009
LFK(+132.04)GHPET LEK	1429.7429	715.8788	2	P02144	y7-H2O	835.4315	835.4308	-0.0007
LFK(+132.04)GHPET LEK	1429.7429	715.8788	2	P02144	y8-H2O	892.4529	892.4523	-0.0006
LFK(+132.04)GHPET LEK	1429.7429	715.8788	2	P02144	y1-NH3	130.0858	130.0858	0
LFK(+132.04)GHPET LEK	1429.7429	715.8784	2	P02144	b2	261.1591	261.1603	0.0012
LFK(+132.04)GHPET LEK	1429.7429	715.8784	2	P02144	b2-H2O	243.1334	243.1497	0.0163
LFK(+132.04)GHPET LEK	1429.7429	715.8784	2	P02144	b6-NH3	795.3954	795.4014	0.006
LFK(+132.04)GHPET	1429.7429	715.8784	2	P02144	b3[2+]	261.1591	261.1476	-0.0115

LEK								
LFK(+132.04)GHPET LEK	1429.7429	715.8784	2	P02144	y1	147.1125	147.1128	0.0003
LFK(+132.04)GHPET LEK	1429.7429	715.8784	2	P02144	y2	276.1548	276.1554	0.0006
LFK(+132.04)GHPET LEK	1429.7429	715.8784	2	P02144	y3	389.2386	389.2394	0.0008
LFK(+132.04)GHPET LEK	1429.7429	715.8784	2	P02144	y4	490.286	490.2871	0.0011
LFK(+132.04)GHPET LEK	1429.7429	715.8784	2	P02144	y5	619.3286	619.3297	0.0012
LFK(+132.04)GHPET LEK	1429.7429	715.8784	2	P02144	y6	716.3802	716.3824	0.0022
LFK(+132.04)GHPET LEK	1429.7429	715.8784	2	P02144	y7	853.438	853.4413	0.0034
LFK(+132.04)GHPET LEK	1429.7429	715.8784	2	P02144	y8	910.4598	910.4628	0.003
LFK(+132.04)GHPET LEK	1429.7429	715.8784	2	P02144	y9	1170.600 7	1170.5978	-0.0029
LFK(+132.04)GHPET LEK	1429.7429	715.8784	2	P02144	y1-H2O	129.102	129.1022	0.0003
LFK(+132.04)GHPET LEK	1429.7429	715.8784	2	P02144	y2-H2O	258.1443	258.1448	0.0005
LFK(+132.04)GHPET LEK	1429.7429	715.8784	2	P02144	y6-H2O	698.3694	698.3719	0.0025
LFK(+132.04)GHPET LEK	1429.7429	715.8784	2	P02144	y7-H2O	835.4271	835.4308	0.0037
LFK(+132.04)GHPET LEK	1429.7429	715.8784	2	P02144	y8-H2O	892.4507	892.4523	0.0015
LFK(+132.04)GHPET LEK	1429.7429	715.8784	2	P02144	y9-H2O	1152.601 3	1152.5873	-0.014
LFK(+132.04)GHPET LEK	1429.7429	715.8784	2	P02144	y1-NH3	130.086	130.0858	-0.0002
LFK(+132.04)GHPET LEK	1429.7429	715.8784	2	P02144	y3-NH3	372.2292	372.2125	-0.0168
LFK(+132.04)GHPET LEK	1429.7429	715.8784	2	P02144	y7[2+]	427.2237	427.2207	-0.003
LFK(+132.04)GHPET LEK	1429.7429	715.8784	2	P02144	y8[2+]	455.7361	455.7314	-0.0047
LFK(+132.04)GHPET LEK	1429.7429	715.8785	2	P02144	b5-NH3	698.3676	698.3486	-0.019
LFK(+132.04)GHPET LEK	1429.7429	715.8785	2	P02144	y1	147.1121	147.1128	0.0007
LFK(+132.04)GHPET	1429.7429	715.8785	2	P02144	y2	276.1551	276.1554	0.0002

LEK								
LFK(+132.04)GHPET LEK	1429.7429	715.8785	2	P02144	y4	490.2854	490.2871	0.0017
LFK(+132.04)GHPET LEK	1429.7429	715.8785	2	P02144	y6	716.3818	716.3824	0.0007
LFK(+132.04)GHPET LEK	1429.7429	715.8785	2	P02144	y7	853.4426	853.4413	-0.0013
LFK(+132.04)GHPET LEK	1429.7429	715.8785	2	P02144	y8	910.4641	910.4628	-0.0012
LFK(+132.04)GHPET LEK	1429.7429	715.8785	2	P02144	y1-H2O	129.1021	129.1022	0.0002
LFK(+132.04)GHPET LEK	1429.7429	715.8785	2	P02144	y6-H2O	698.3676	698.3719	0.0043
LFK(+132.04)GHPET LEK	1429.7429	715.8784	2	P02144	b2	261.1588	261.1603	0.0015
LFK(+132.04)GHPET LEK	1429.7429	715.8784	2	P02144	b2-H2O	243.1337	243.1497	0.016
LFK(+132.04)GHPET LEK	1429.7429	715.8784	2	P02144	b3[2+]	261.1588	261.1476	-0.0111
LFK(+132.04)GHPET LEK	1429.7429	715.8784	2	P02144	y1	147.1125	147.1128	0.0003
LFK(+132.04)GHPET LEK	1429.7429	715.8784	2	P02144	y2	276.1549	276.1554	0.0004
LFK(+132.04)GHPET LEK	1429.7429	715.8784	2	P02144	y3	389.2391	389.2394	0.0003
LFK(+132.04)GHPET LEK	1429.7429	715.8784	2	P02144	y4	490.2862	490.2871	0.0009
LFK(+132.04)GHPET LEK	1429.7429	715.8784	2	P02144	y5	619.3289	619.3297	0.0009
LFK(+132.04)GHPET LEK	1429.7429	715.8784	2	P02144	y6	716.381	716.3824	0.0015
LFK(+132.04)GHPET LEK	1429.7429	715.8784	2	P02144	y7	853.4386	853.4413	0.0027
LFK(+132.04)GHPET LEK	1429.7429	715.8784	2	P02144	y8	910.4609	910.4628	0.002
LFK(+132.04)GHPET LEK	1429.7429	715.8784	2	P02144	y1-H2O	129.102	129.1022	0.0002
LFK(+132.04)GHPET LEK	1429.7429	715.8784	2	P02144	y2-H2O	258.1443	258.1448	0.0005
LFK(+132.04)GHPET LEK	1429.7429	715.8784	2	P02144	y6-H2O	698.3703	698.3719	0.0016
LFK(+132.04)GHPET LEK	1429.7429	715.8784	2	P02144	y7-H2O	835.4309	835.4308	-0.0001
LFK(+132.04)GHPET	1429.7429	715.8784	2	P02144	y8-H2O	892.4509	892.4523	0.0014

LEK								
LFK(+132.04)GHPET LEK	1429.7429	715.8784	2	P02144	y1-NH3	130.086	130.0858	-0.0002
LFK(+132.04)GHPET LEK	1429.7429	715.8783	2	P02144	y1	147.1125	147.1128	0.0003
LFK(+132.04)GHPET LEK	1429.7429	715.8783	2	P02144	y2	276.1559	276.1554	-0.0006
LFK(+132.04)GHPET LEK	1429.7429	715.8783	2	P02144	y3	389.2363	389.2394	0.0031
LFK(+132.04)GHPET LEK	1429.7429	715.8783	2	P02144	y4	490.2863	490.2871	0.0008
LFK(+132.04)GHPET LEK	1429.7429	715.8783	2	P02144	y6	716.3812	716.3824	0.0012
LFK(+132.04)GHPET LEK	1429.7429	715.8783	2	P02144	y8	910.4604	910.4628	0.0024
LFK(+132.04)GHPET LEK	1429.7429	715.8783	2	P02144	y1-H2O	129.102	129.1022	0.0003
LFK(+132.04)GHPET LEK	1429.7429	715.8783	2	P02144	y1-NH3	130.086	130.0858	-0.0002
LFK(+132.04)GHPET LEK	1429.7429	715.8788	2	P02144	y1	147.1128	147.1128	0
LFK(+132.04)GHPET LEK	1429.7429	715.8788	2	P02144	y3	389.2379	389.2394	0.0015
LFK(+132.04)GHPET LEK	1429.7429	715.8788	2	P02144	y4	490.2853	490.2871	0.0018
LFK(+132.04)GHPET LEK	1429.7429	715.8788	2	P02144	y6	716.3806	716.3824	0.0019
LFK(+132.04)GHPET LEK	1429.7429	715.8788	2	P02144	y8	910.4568	910.4628	0.006
LFK(+132.04)GHPET LEK	1429.7429	715.8788	2	P02144	y1-H2O	129.1021	129.1022	0.0002
LFK(+132.04)GHPET LEK	1429.7429	715.8788	2	P02144	y2-H2O	258.1447	258.1448	0.0001
LFK(+132.04)GHPET LEK	1429.7429	715.8788	2	P02144	y1-NH3	130.0858	130.0858	0
LFK(+132.04)GHPET LEK	1429.7429	715.8777	2	P02144	y1	147.1125	147.1128	0.0003
LFK(+132.04)GHPET LEK	1429.7429	715.8777	2	P02144	y2	276.1549	276.1554	0.0004
LFK(+132.04)GHPET LEK	1429.7429	715.8777	2	P02144	y3	389.2395	389.2394	-0.0001
LFK(+132.04)GHPET LEK	1429.7429	715.8777	2	P02144	y4	490.2871	490.2871	0
LFK(+132.04)GHPET	1429.7429	715.8777	2	P02144	y6	716.3821	716.3824	0.0003

LEK								
LFK(+132.04)GHPET LEK	1429.7429	715.8777	2	P02144	y7	853.4407	853.4413	0.0007
LFK(+132.04)GHPET LEK	1429.7429	715.8777	2	P02144	y8	910.4648	910.4628	-0.002
LFK(+132.04)GHPET LEK	1429.7429	715.8777	2	P02144	y1-H2O	129.1021	129.1022	0.0002
LFK(+132.04)GHPET LEK	1429.7429	715.8777	2	P02144	y2-H2O	258.1446	258.1448	0.0002
LFK(+132.04)GHPET LEK	1429.7429	715.8777	2	P02144	y6-H2O	698.3724	698.3719	-0.0005
LFK(+132.04)GHPET LEK	1429.7429	715.8777	2	P02144	y1-NH3	130.086	130.0858	-0.0002
LFK(+132.04)GHPET LEK	1429.7429	715.8777	2	P02144	y2-NH3	259.1284	259.1284	0
LFK(+132.04)GHPET LEK	1429.7429	477.588	3	P02144	b2	261.1592	261.1603	0.001
LFK(+132.04)GHPET LEK	1429.7429	477.588	3	P02144	b2-H2O	243.1332	243.1497	0.0165
LFK(+132.04)GHPET LEK	1429.7429	477.588	3	P02144	b5-H2O	697.3554	697.3651	0.0096
LFK(+132.04)GHPET LEK	1429.7429	477.588	3	P02144	b7-H2O	923.4412	923.4604	0.0192
LFK(+132.04)GHPET LEK	1429.7429	477.588	3	P02144	b3[2+]	261.1592	261.1476	-0.0116
LFK(+132.04)GHPET LEK	1429.7429	477.588	3	P02144	y1	147.1125	147.1128	0.0003
LFK(+132.04)GHPET LEK	1429.7429	477.588	3	P02144	y2	276.1546	276.1554	0.0007
LFK(+132.04)GHPET LEK	1429.7429	477.588	3	P02144	y3	389.2383	389.2394	0.0011
LFK(+132.04)GHPET LEK	1429.7429	477.588	3	P02144	y4	490.2858	490.2871	0.0013
LFK(+132.04)GHPET LEK	1429.7429	477.588	3	P02144	y5	619.3278	619.3297	0.0019
LFK(+132.04)GHPET LEK	1429.7429	477.588	3	P02144	y6	716.3801	716.3824	0.0024
LFK(+132.04)GHPET LEK	1429.7429	477.588	3	P02144	y7	853.4397	853.4413	0.0016
LFK(+132.04)GHPET LEK	1429.7429	477.588	3	P02144	y8	910.4608	910.4628	0.0021
LFK(+132.04)GHPET LEK	1429.7429	477.588	3	P02144	y1-H2O	129.1019	129.1022	0.0003
LFK(+132.04)GHPET	1429.7429	477.588	3	P02144	y2-H2O	258.1442	258.1448	0.0006

LEK								
LFK(+132.04)GHPET LEK	1429.7429	477.588	3	P02144	y5-H2O	601.317	601.3192	0.0022
LFK(+132.04)GHPET LEK	1429.7429	477.588	3	P02144	y6-H2O	698.3702	698.3719	0.0017
LFK(+132.04)GHPET LEK	1429.7429	477.588	3	P02144	y8-H2O	892.4521	892.4523	0.0002
LFK(+132.04)GHPET LEK	1429.7429	477.588	3	P02144	y1-NH3	130.086	130.0858	-0.0002
LFK(+132.04)GHPET LEK	1429.7429	358.4427	4	P02144	b2-H2O	243.1337	243.1497	0.016
LFK(+132.04)GHPET LEK	1429.7429	358.4427	4	P02144	y1	147.1126	147.1128	0.0002
LFK(+132.04)GHPET LEK	1429.7429	358.4427	4	P02144	y2	276.1551	276.1554	0.0003
LFK(+132.04)GHPET LEK	1429.7429	358.4427	4	P02144	y3	389.2389	389.2394	0.0005
LFK(+132.04)GHPET LEK	1429.7429	358.4427	4	P02144	y4	490.2865	490.2871	0.0006
LFK(+132.04)GHPET LEK	1429.7429	358.4427	4	P02144	y5	619.3285	619.3297	0.0012
LFK(+132.04)GHPET LEK	1429.7429	358.4427	4	P02144	y6	716.3809	716.3824	0.0015
LFK(+132.04)GHPET LEK	1429.7429	358.4427	4	P02144	y8	910.4658	910.4628	-0.003
LFK(+132.04)GHPET LEK	1429.7429	358.4427	4	P02144	y1-H2O	129.1021	129.1022	0.0002
LFK(+132.04)GHPET LEK	1429.7429	358.4427	4	P02144	y2-H2O	258.1445	258.1448	0.0003
LFK(+132.04)GHPET LEK	1429.7429	358.4427	4	P02144	y4-H2O	472.2763	472.2766	0.0003
LFK(+132.04)GHPET LEK	1429.7429	358.4427	4	P02144	y5-H2O	601.3183	601.3192	0.0009
LFK(+132.04)GHPET LEK	1429.7429	358.4427	4	P02144	y6-H2O	698.3707	698.3719	0.0012
LFK(+132.04)GHPET LEK	1429.7429	358.4427	4	P02144	y1-NH3	130.0861	130.0858	-0.0003
LFK(+132.04)GHPET LEK	1429.7429	358.4427	4	P02144	y3[2+]	195.1125	195.1197	0.0072
LFK(+132.04)GHPET LEK	1429.7429	477.588	3	P02144	b2	261.1587	261.1603	0.0016
LFK(+132.04)GHPET LEK	1429.7429	477.588	3	P02144	b2-H2O	243.1337	243.1497	0.0161
LFK(+132.04)GHPET	1429.7429	477.588	3	P02144	b7-H2O	923.446	923.4604	0.0144

LEK								
LFK(+132.04)GHPET LEK	1429.7429	477.588	3	P02144	b3[2+]	261.1587	261.1476	-0.011
LFK(+132.04)GHPET LEK	1429.7429	477.588	3	P02144	y1	147.1125	147.1128	0.0002
LFK(+132.04)GHPET LEK	1429.7429	477.588	3	P02144	y2	276.1548	276.1554	0.0005
LFK(+132.04)GHPET LEK	1429.7429	477.588	3	P02144	y3	389.2382	389.2394	0.0012
LFK(+132.04)GHPET LEK	1429.7429	477.588	3	P02144	y4	490.2862	490.2871	0.0009
LFK(+132.04)GHPET LEK	1429.7429	477.588	3	P02144	y5	619.3281	619.3297	0.0016
LFK(+132.04)GHPET LEK	1429.7429	477.588	3	P02144	y6	716.3803	716.3824	0.0021
LFK(+132.04)GHPET LEK	1429.7429	477.588	3	P02144	y8	910.4598	910.4628	0.003
LFK(+132.04)GHPET LEK	1429.7429	477.588	3	P02144	y1-H2O	129.102	129.1022	0.0002
LFK(+132.04)GHPET LEK	1429.7429	477.588	3	P02144	y2-H2O	258.1443	258.1448	0.0005
LFK(+132.04)GHPET LEK	1429.7429	477.588	3	P02144	y6-H2O	698.3687	698.3719	0.0032
LFK(+132.04)GHPET LEK	1429.7429	477.588	3	P02144	y8-H2O	892.4509	892.4523	0.0013
LFK(+132.04)GHPET LEK	1429.7429	477.588	3	P02144	y1-NH3	130.086	130.0858	-0.0003
LFK(+132.04)GHPET LEK	1429.7429	477.588	3	P02144	y5-NH3	602.3153	602.3027	-0.0126
LFK(+132.04)GHPET LEK	1429.7429	477.588	3	P02144	y6-NH3	699.3717	699.3555	-0.0162
LFK(+132.04)GHPET LEK	1429.7429	477.588	3	P02144	y8-NH3	893.454	893.4359	-0.0182
LFK(+132.04)GHPET LEK	1429.7429	477.588	3	P02144	y3[2+]	195.1107	195.1197	0.009
LFK(+132.04)GHPET LEK	1429.7429	715.878	2	P02144	y1	147.1125	147.1128	0.0003
LFK(+132.04)GHPET LEK	1429.7429	715.878	2	P02144	y2	276.1559	276.1554	-0.0006
LFK(+132.04)GHPET LEK	1429.7429	715.878	2	P02144	y3	389.2363	389.2394	0.0031
LFK(+132.04)GHPET LEK	1429.7429	715.878	2	P02144	y4	490.2863	490.2871	0.0008
LFK(+132.04)GHPET	1429.7429	715.878	2	P02144	y6	716.3812	716.3824	0.0012

LEK								
LFK(+132.04)GHPET LEK	1429.7429	715.878	2	P02144	y8	910.4604	910.4628	0.0024
LFK(+132.04)GHPET LEK	1429.7429	715.878	2	P02144	y1-H2O	129.102	129.1022	0.0003
LFK(+132.04)GHPET LEK	1429.7429	715.878	2	P02144	y1-NH3	130.086	130.0858	-0.0002
LFK(+132.04)GHPET LEK	1429.7429	477.588	3	P02144	b2-H2O	243.1337	243.1497	0.0161
LFK(+132.04)GHPET LEK	1429.7429	477.588	3	P02144	y1	147.1125	147.1128	0.0003
LFK(+132.04)GHPET LEK	1429.7429	477.588	3	P02144	y2	276.1549	276.1554	0.0004
LFK(+132.04)GHPET LEK	1429.7429	477.588	3	P02144	y3	389.2388	389.2394	0.0007
LFK(+132.04)GHPET LEK	1429.7429	477.588	3	P02144	y4	490.2865	490.2871	0.0006
LFK(+132.04)GHPET LEK	1429.7429	477.588	3	P02144	y5	619.3281	619.3297	0.0016
LFK(+132.04)GHPET LEK	1429.7429	477.588	3	P02144	y6	716.3815	716.3824	0.0009
LFK(+132.04)GHPET LEK	1429.7429	477.588	3	P02144	y8	910.4656	910.4628	-0.0028
LFK(+132.04)GHPET LEK	1429.7429	477.588	3	P02144	y1-H2O	129.102	129.1022	0.0002
LFK(+132.04)GHPET LEK	1429.7429	477.588	3	P02144	y2-H2O	258.1443	258.1448	0.0005
LFK(+132.04)GHPET LEK	1429.7429	477.588	3	P02144	y5-H2O	601.3194	601.3192	-0.0002
LFK(+132.04)GHPET LEK	1429.7429	477.588	3	P02144	y8-H2O	892.4498	892.4523	0.0025
LFK(+132.04)GHPET LEK	1429.7429	477.588	3	P02144	y1-NH3	130.086	130.0858	-0.0003
LFK(+132.04)GHPET LEK	1429.7429	715.8787	2	P02144	y1	147.1124	147.1128	0.0004
LFK(+132.04)GHPET LEK	1429.7429	715.8787	2	P02144	y2	276.1558	276.1554	-0.0005
LFK(+132.04)GHPET LEK	1429.7429	715.8787	2	P02144	y3	389.2398	389.2394	-0.0003
LFK(+132.04)GHPET LEK	1429.7429	715.8787	2	P02144	y4	490.2858	490.2871	0.0013
LFK(+132.04)GHPET LEK	1429.7429	715.8787	2	P02144	y6	716.3832	716.3824	-0.0007
LFK(+132.04)GHPET	1429.7429	715.8787	2	P02144	y1-H2O	129.102	129.1022	0.0002

LEK								
LFK(+132.04)GHPET LEK	1429.7429	715.8787	2	P02144	y2-H2O	258.1444	258.1448	0.0004
LFK(+132.04)GHPET LEK	1429.7429	477.5881	3	P02144	b2-H2O	243.1341	243.1497	0.0156
LFK(+132.04)GHPET LEK	1429.7429	477.5881	3	P02144	b3[2+]	261.1383	261.1476	0.0093
LFK(+132.04)GHPET LEK	1429.7429	477.5881	3	P02144	y1	147.1126	147.1128	0.0002
LFK(+132.04)GHPET LEK	1429.7429	477.5881	3	P02144	y2	276.1554	276.1554	0
LFK(+132.04)GHPET LEK	1429.7429	477.5881	3	P02144	y3	389.2384	389.2394	0.001
LFK(+132.04)GHPET LEK	1429.7429	477.5881	3	P02144	y4	490.2872	490.2871	-0.0001
LFK(+132.04)GHPET LEK	1429.7429	477.5881	3	P02144	y5	619.3297	619.3297	0.0001
LFK(+132.04)GHPET LEK	1429.7429	477.5881	3	P02144	y6	716.3829	716.3824	-0.0005
LFK(+132.04)GHPET LEK	1429.7429	477.5881	3	P02144	y8	910.4598	910.4628	0.003
LFK(+132.04)GHPET LEK	1429.7429	477.5881	3	P02144	y1-H2O	129.102	129.1022	0.0002
LFK(+132.04)GHPET LEK	1429.7429	477.5881	3	P02144	y2-H2O	258.1444	258.1448	0.0004
LFK(+132.04)GHPET LEK	1429.7429	477.5881	3	P02144	y6-H2O	698.3729	698.3719	-0.001
LFK(+132.04)GHPET LEK	1429.7429	477.5881	3	P02144	y1-NH3	130.086	130.0858	-0.0003
LFK(+132.04)GHPET LEK	1429.7429	715.877	2	P02144	b5	715.362	715.3756	0.0136
LFK(+132.04)GHPET LEK	1429.7429	715.877	2	P02144	y1	147.1126	147.1128	0.0002
LFK(+132.04)GHPET LEK	1429.7429	715.877	2	P02144	y4	490.285	490.2871	0.0021
LFK(+132.04)GHPET LEK	1429.7429	715.877	2	P02144	y6	716.3819	716.3824	0.0005
LFK(+132.04)GHPET LEK	1429.7429	715.877	2	P02144	y8	910.4615	910.4628	0.0013
LFK(+132.04)GHPET LEK	1429.7429	715.877	2	P02144	y1-H2O	129.1021	129.1022	0.0002
LFK(+132.04)GHPET LEK	1429.7429	715.877	2	P02144	y2-H2O	258.1439	258.1448	0.0009
LFK(+132.04)GHPET	1429.7429	715.877	2	P02144	y6-H2O	698.3732	698.3719	-0.0013

LEK								
LFK(+132.04)GHPET LEK	1429.7429	477.5879	3	P02144	y1	147.1126	147.1128	0.0002
LFK(+132.04)GHPET LEK	1429.7429	477.5879	3	P02144	y2	276.1547	276.1554	0.0007
LFK(+132.04)GHPET LEK	1429.7429	477.5879	3	P02144	y4	490.2868	490.2871	0.0003
LFK(+132.04)GHPET LEK	1429.7429	477.5879	3	P02144	y5	619.3288	619.3297	0.0009
LFK(+132.04)GHPET LEK	1429.7429	477.5879	3	P02144	y6	716.3802	716.3824	0.0022
LFK(+132.04)GHPET LEK	1429.7429	477.5879	3	P02144	y8	910.455	910.4628	0.0078
LFK(+132.04)GHPET LEK	1429.7429	477.5879	3	P02144	y1-H2O	129.102	129.1022	0.0002
LFK(+132.04)GHPET LEK	1429.7429	477.5879	3	P02144	y2-H2O	258.1438	258.1448	0.001
LFK(+132.04)GHPET LEK	1429.7429	477.5879	3	P02144	y1-NH3	130.086	130.0858	-0.0002
GHHEAEIK(+132.04) PLAQSHATK	1984.9944	993.5029	2	P02144	b2	195.087	195.0882	0.0012
GHHEAEIK(+132.04) PLAQSHATK	1984.9944	993.5029	2	P02144	b3	332.1456	332.1471	0.0015
GHHEAEIK(+132.04) PLAQSHATK	1984.9944	993.5029	2	P02144	b4	461.1879	461.1897	0.0018
GHHEAEIK(+132.04) PLAQSHATK	1984.9944	993.5029	2	P02144	b5	532.2253	532.2268	0.0015
GHHEAEIK(+132.04) PLAQSHATK	1984.9944	993.5029	2	P02144	b6	661.2671	661.2694	0.0023
GHHEAEIK(+132.04) PLAQSHATK	1984.9944	993.5029	2	P02144	b7	774.3525	774.3535	0.001
GHHEAEIK(+132.04) PLAQSHATK	1984.9944	993.5029	2	P02144	b3-H2O	314.1353	314.1365	0.0013
GHHEAEIK(+132.04) PLAQSHATK	1984.9944	993.5029	2	P02144	b4-H2O	443.1789	443.1791	0.0002
GHHEAEIK(+132.04) PLAQSHATK	1984.9944	993.5029	2	P02144	b5-H2O	514.2141	514.2162	0.0022
GHHEAEIK(+132.04) PLAQSHATK	1984.9944	993.5029	2	P02144	b6-H2O	643.2568	643.2589	0.002
GHHEAEIK(+132.04) PLAQSHATK	1984.9944	993.5029	2	P02144	b7-H2O	756.3328	756.3429	0.0101
GHHEAEIK(+132.04) PLAQSHATK	1984.9944	993.5029	2	P02144	b2-NH3	178.0608	178.0612	0.0004
GHHEAEIK(+132.04)	1984.9944	993.5029	2	P02144	y2	248.1602	248.1605	0.0003

PLAQSHATK								
GHHEAEIK(+132.04) PLAQSHATK	1984.9944	993.5029	2	P02144	y3	319.1966	319.1976	0.001
GHHEAEIK(+132.04) PLAQSHATK	1984.9944	993.5029	2	P02144	y4	456.2558	456.2565	0.0006
GHHEAEIK(+132.04) PLAQSHATK	1984.9944	993.5029	2	P02144	y5	543.2875	543.2885	0.001
GHHEAEIK(+132.04) PLAQSHATK	1984.9944	993.5029	2	P02144	y6	671.3473	671.3471	-0.0002
GHHEAEIK(+132.04) PLAQSHATK	1984.9944	993.5029	2	P02144	y7	742.3799	742.3842	0.0043
GHHEAEIK(+132.04) PLAQSHATK	1984.9944	993.5029	2	P02144	y8	855.4664	855.4683	0.0019
GHHEAEIK(+132.04) PLAQSHATK	1984.9944	993.5029	2	P02144	y9	952.519	952.521	0.002
GHHEAEIK(+132.04) PLAQSHATK	1984.9944	993.5029	2	P02144	y9-H2O	934.5077	934.5104	0.0027
GHHEAEIK(+132.04) PLAQSHATK	1984.9944	398.0054	5	P02144	b2	195.0871	195.0882	0.0011
GHHEAEIK(+132.04) PLAQSHATK	1984.9944	398.0054	5	P02144	b4	461.189	461.1897	0.0007
GHHEAEIK(+132.04) PLAQSHATK	1984.9944	398.0054	5	P02144	b5	532.226	532.2268	0.0008
GHHEAEIK(+132.04) PLAQSHATK	1984.9944	398.0054	5	P02144	b6	661.2674	661.2694	0.002
GHHEAEIK(+132.04) PLAQSHATK	1984.9944	398.0054	5	P02144	b3-H2O	314.1356	314.1365	0.001
GHHEAEIK(+132.04) PLAQSHATK	1984.9944	398.0054	5	P02144	b4-H2O	443.1772	443.1791	0.0019
GHHEAEIK(+132.04) PLAQSHATK	1984.9944	398.0054	5	P02144	b3[2+]	166.5768	166.5735	-0.0032
GHHEAEIK(+132.04) PLAQSHATK	1984.9944	398.0054	5	P02144	b4[2+]	231.0977	231.0948	-0.0029
GHHEAEIK(+132.04) PLAQSHATK	1984.9944	398.0054	5	P02144	b5[2+]	266.6163	266.6134	-0.0029
GHHEAEIK(+132.04) PLAQSHATK	1984.9944	398.0054	5	P02144	b10[2+]	622.817	622.8126	-0.0044
GHHEAEIK(+132.04) PLAQSHATK	1984.9944	398.0054	5	P02144	y2	248.16	248.1605	0.0004
GHHEAEIK(+132.04) PLAQSHATK	1984.9944	398.0054	5	P02144	y3	319.1971	319.1976	0.0005
GHHEAEIK(+132.04) PLAQSHATK	1984.9944	398.0054	5	P02144	y4	456.2555	456.2565	0.0009
GHHEAEIK(+132.04)	1984.9944	398.0054	5	P02144	y5	543.2874	543.2885	0.0011

PLAQSHATK								
GHHEAEIK(+132.04) PLAQSHATK	1984.9944	398.0054	5	P02144	y6	671.3454	671.3471	0.0017
GHHEAEIK(+132.04) PLAQSHATK	1984.9944	398.0054	5	P02144	y7	742.3823	742.3842	0.002
GHHEAEIK(+132.04) PLAQSHATK	1984.9944	398.0054	5	P02144	y8	855.4673	855.4683	0.001
GHHEAEIK(+132.04) PLAQSHATK	1984.9944	398.0054	5	P02144	y9	952.5187	952.521	0.0023
GHHEAEIK(+132.04) PLAQSHATK	1984.9944	398.0054	5	P02144	y2-H2O	230.1496	230.1499	0.0003
GHHEAEIK(+132.04) PLAQSHATK	1984.9944	398.0054	5	P02144	y4-H2O	438.2437	438.2459	0.0022
GHHEAEIK(+132.04) PLAQSHATK	1984.9944	398.0054	5	P02144	y5-H2O	525.2753	525.278	0.0026
GHHEAEIK(+132.04) PLAQSHATK	1984.9944	398.0054	5	P02144	y6-H2O	653.3369	653.3365	-0.0003
GHHEAEIK(+132.04) PLAQSHATK	1984.9944	398.0054	5	P02144	y7-H2O	724.3713	724.3737	0.0023
GHHEAEIK(+132.04) PLAQSHATK	1984.9944	398.0054	5	P02144	y6-NH3	654.322	654.3201	-0.0019
GHHEAEIK(+132.04) PLAQSHATK	1984.9944	398.0054	5	P02144	y7-NH3	725.3637	725.3572	-0.0065
GHHEAEIK(+132.04) PLAQSHATK	1984.9944	398.0054	5	P02144	y9-NH3	935.5002	935.494	-0.0062
GHHEAEIK(+132.04) PLAQSHATK	1984.9944	398.0054	5	P02144	y4[2+]	228.6314	228.6282	-0.0032
GHHEAEIK(+132.04) PLAQSHATK	1984.9944	398.0054	5	P02144	y5[2+]	272.1476	272.1443	-0.0033
GHHEAEIK(+132.04) PLAQSHATK	1984.9944	398.0054	5	P02144	y8[2+]	428.2376	428.2341	-0.0035
GHHEAEIK(+132.04) PLAQSHATK	1984.9944	497.2552	4	P02144	b2	195.0872	195.0882	0.001
GHHEAEIK(+132.04) PLAQSHATK	1984.9944	497.2552	4	P02144	b4	461.188	461.1897	0.0017
GHHEAEIK(+132.04) PLAQSHATK	1984.9944	497.2552	4	P02144	b5	532.2249	532.2268	0.002
GHHEAEIK(+132.04) PLAQSHATK	1984.9944	497.2552	4	P02144	b6	661.2669	661.2694	0.0025
GHHEAEIK(+132.04) PLAQSHATK	1984.9944	497.2552	4	P02144	b7	774.3519	774.3535	0.0015
GHHEAEIK(+132.04) PLAQSHATK	1984.9944	497.2552	4	P02144	b3-H2O	314.1353	314.1365	0.0012
GHHEAEIK(+132.04)	1984.9944	497.2552	4	P02144	b2-NH3	178.0607	178.0612	0.0005

PLAQSHATK								
GHHEAEIK(+132.04) PLAQSHATK	1984.9944	497.2552	4	P02144	y2	248.16	248.1605	0.0004
GHHEAEIK(+132.04) PLAQSHATK	1984.9944	497.2552	4	P02144	y3	319.1969	319.1976	0.0007
GHHEAEIK(+132.04) PLAQSHATK	1984.9944	497.2552	4	P02144	y4	456.2554	456.2565	0.0011
GHHEAEIK(+132.04) PLAQSHATK	1984.9944	497.2552	4	P02144	y5	543.2873	543.2885	0.0012
GHHEAEIK(+132.04) PLAQSHATK	1984.9944	497.2552	4	P02144	y6	671.3446	671.3471	0.0025
GHHEAEIK(+132.04) PLAQSHATK	1984.9944	497.2552	4	P02144	y7	742.3819	742.3842	0.0023
GHHEAEIK(+132.04) PLAQSHATK	1984.9944	497.2552	4	P02144	y8	855.465	855.4683	0.0032
GHHEAEIK(+132.04) PLAQSHATK	1984.9944	497.2552	4	P02144	y9	952.5175	952.521	0.0035
GHHEAEIK(+132.04) PLAQSHATK	1984.9944	497.2552	4	P02144	y2-H2O	230.1496	230.1499	0.0003
GHHEAEIK(+132.04) PLAQSHATK	1984.9944	497.2552	4	P02144	y3-H2O	301.1872	301.187	-0.0002
GHHEAEIK(+132.04) PLAQSHATK	1984.9944	497.2552	4	P02144	y5-H2O	525.2754	525.278	0.0026
GHHEAEIK(+132.04) PLAQSHATK	1984.9944	497.2552	4	P02144	y6-H2O	653.3356	653.3365	0.001
GHHEAEIK(+132.04) PLAQSHATK	1984.9944	497.2552	4	P02144	y7-H2O	724.3733	724.3737	0.0004
GHHEAEIK(+132.04) PLAQSHATK	1984.9944	497.2552	4	P02144	y9-H2O	934.5184	934.5104	-0.0079
GHHEAEIK(+132.04) PLAQSHATK	1984.9944	497.2552	4	P02144	y6-NH3	654.3206	654.3201	-0.0004
GHHEAEIK(+132.04) PLAQSHATK	1984.9944	497.2552	4	P02144	y7-NH3	725.3631	725.3572	-0.0059
GHHEAEIK(+132.04) PLAQSHATK	1984.9944	497.2552	4	P02144	y8-NH3	838.4538	838.4413	-0.0125
GHHEAEIK(+132.04) PLAQSHATK	1984.9944	497.2552	4	P02144	y9-NH3	935.5021	935.494	-0.0081
GHHEAEIK(+132.04) PLAQSHATK	1984.9944	497.2552	4	P02144	y7[2+]	371.6949	371.6921	-0.0028
GHHEAEIK(+132.04) PLAQSHATK	1984.9944	662.671	3	P02144	b2	195.0873	195.0882	0.0009
GHHEAEIK(+132.04) PLAQSHATK	1984.9944	662.671	3	P02144	b3	332.146	332.1471	0.0011
GHHEAEIK(+132.04)	1984.9944	662.671	3	P02144	b4	461.1882	461.1897	0.0015

PLAQSHATK								
GHHEAEIK(+132.04) PLAQSHATK	1984.9944	662.671	3	P02144	b5	532.225	532.2268	0.0018
GHHEAEIK(+132.04) PLAQSHATK	1984.9944	662.671	3	P02144	b6	661.2674	661.2694	0.002
GHHEAEIK(+132.04) PLAQSHATK	1984.9944	662.671	3	P02144	b7	774.3521	774.3535	0.0013
GHHEAEIK(+132.04) PLAQSHATK	1984.9944	662.671	3	P02144	b3-H2O	314.1355	314.1365	0.001
GHHEAEIK(+132.04) PLAQSHATK	1984.9944	662.671	3	P02144	b4-H2O	443.1785	443.1791	0.0006
GHHEAEIK(+132.04) PLAQSHATK	1984.9944	662.671	3	P02144	b2-NH3	178.0608	178.0612	0.0004
GHHEAEIK(+132.04) PLAQSHATK	1984.9944	662.671	3	P02144	b3-NH3	315.1385	315.1201	-0.0184
GHHEAEIK(+132.04) PLAQSHATK	1984.9944	662.671	3	P02144	b6-NH3	644.2597	644.2424	-0.0173
GHHEAEIK(+132.04) PLAQSHATK	1984.9944	662.671	3	P02144	b6[2+]	331.1375	331.1347	-0.0028
GHHEAEIK(+132.04) PLAQSHATK	1984.9944	662.671	3	P02144	y2	248.1601	248.1605	0.0004
GHHEAEIK(+132.04) PLAQSHATK	1984.9944	662.671	3	P02144	y3	319.1971	319.1976	0.0005
GHHEAEIK(+132.04) PLAQSHATK	1984.9944	662.671	3	P02144	y4	456.2552	456.2565	0.0012
GHHEAEIK(+132.04) PLAQSHATK	1984.9944	662.671	3	P02144	y5	543.2878	543.2885	0.0007
GHHEAEIK(+132.04) PLAQSHATK	1984.9944	662.671	3	P02144	y6	671.3461	671.3471	0.001
GHHEAEIK(+132.04) PLAQSHATK	1984.9944	662.671	3	P02144	y7	742.3823	742.3842	0.0019
GHHEAEIK(+132.04) PLAQSHATK	1984.9944	662.671	3	P02144	y8	855.465	855.4683	0.0033
GHHEAEIK(+132.04) PLAQSHATK	1984.9944	662.671	3	P02144	y9	952.5188	952.521	0.0022
GHHEAEIK(+132.04) PLAQSHATK	1984.9944	662.671	3	P02144	y2-H2O	230.1497	230.1499	0.0002
GHHEAEIK(+132.04) PLAQSHATK	1984.9944	662.671	3	P02144	y3-H2O	301.1867	301.187	0.0003
GHHEAEIK(+132.04) PLAQSHATK	1984.9944	662.671	3	P02144	y4-H2O	438.2462	438.2459	-0.0003
GHHEAEIK(+132.04) PLAQSHATK	1984.9944	662.671	3	P02144	y9-H2O	934.5082	934.5104	0.0022
GHHEAEIK(+132.04)	1984.9944	662.671	3	P02144	y9-NH3	935.5074	935.494	-0.0134

PLAQSHATK GHHEAEIK(+132.04) PLAQSHATK	1984.9944	662.671	3	P02144	y10-NH3	1195.6174	1195.629	0.0116
SEDEMK(+132.04)ASEDLK	1512.6478	757.3307	2	P02144	b2	217.0814	217.0824	0.001
SEDEMK(+132.04)ASEDLK	1512.6478	757.3307	2	P02144	b3	332.1082	332.1094	0.0012
SEDEMK(+132.04)ASEDLK	1512.6478	757.3307	2	P02144	b4	461.1509	461.1519	0.0011
SEDEMK(+132.04)ASEDLK	1512.6478	757.3307	2	P02144	b5	592.1906	592.1924	0.0019
SEDEMK(+132.04)ASEDLK	1512.6478	757.3307	2	P02144	b10	1254.4658	1254.4661	0.0002
SEDEMK(+132.04)ASEDLK	1512.6478	757.3307	2	P02144	b2-H2O	199.0714	199.0719	0.0004
SEDEMK(+132.04)ASEDLK	1512.6478	757.3307	2	P02144	b3-H2O	314.0981	314.0988	0.0007
SEDEMK(+132.04)ASEDLK	1512.6478	757.3307	2	P02144	b4-H2O	443.1407	443.1414	0.0007
SEDEMK(+132.04)ASEDLK	1512.6478	757.3307	2	P02144	b5-H2O	574.1797	574.1819	0.0021
SEDEMK(+132.04)ASEDLK	1512.6478	757.3307	2	P02144	b6-H2O	834.3123	834.3168	0.0045
SEDEMK(+132.04)ASEDLK	1512.6478	757.3307	2	P02144	b7-NH3	906.3514	906.3375	-0.0139
SEDEMK(+132.04)ASEDLK	1512.6478	757.3307	2	P02144	b9-NH3	1122.4249	1122.4121	-0.0128
SEDEMK(+132.04)ASEDLK	1512.6478	757.3307	2	P02144	y1	147.1125	147.1128	0.0003
SEDEMK(+132.04)ASEDLK	1512.6478	757.3307	2	P02144	y2	260.1963	260.1968	0.0005
SEDEMK(+132.04)ASEDLK	1512.6478	757.3307	2	P02144	y3	375.223	375.2238	0.0008
SEDEMK(+132.04)ASEDLK	1512.6478	757.3307	2	P02144	y4	504.2656	504.2664	0.0007
SEDEMK(+132.04)ASEDLK	1512.6478	757.3307	2	P02144	y5	591.2977	591.2984	0.0007
SEDEMK(+132.04)ASEDLK	1512.6478	757.3307	2	P02144	y6	662.3344	662.3355	0.0011
SEDEMK(+132.04)ASEDLK	1512.6478	757.3307	2	P02144	y1-H2O	129.102	129.1022	0.0003
SEDEMK(+132.04)ASEDLK	1512.6478	757.3307	2	P02144	y2-H2O	242.1858	242.1863	0.0005

SEDEMK(+132.04)AS EDLK	1512.6478	757.3307	2	P02144	y4-H2O	486.2553	486.2558	0.0005
SEDEMK(+132.04)AS EDLK	1512.6478	757.3307	2	P02144	y5-H2O	573.2876	573.2878	0.0002
SEDEMK(+132.04)AS EDLK	1512.6478	757.3307	2	P02144	y6-H2O	644.3219	644.325	0.0031
SEDEMK(+132.04)AS EDLK	1512.6478	757.3307	2	P02144	y1-NH3	130.086	130.0858	-0.0002
SEDEMK(+132.04)AS EDLK	1512.6478	757.3307	2	P02144	y2-NH3	243.1698	243.1698	0
SEDEMK(+132.04)AS EDLK	1512.6478	757.3307	2	P02144	y9-NH3	1165.534 2	1165.5266	-0.0076
SEDEMK(+132.04)AS EDLK	1512.6478	505.2228	3	P02144	b2	217.0816	217.0824	0.0008
SEDEMK(+132.04)AS EDLK	1512.6478	505.2228	3	P02144	b3	332.1086	332.1094	0.0008
SEDEMK(+132.04)AS EDLK	1512.6478	505.2228	3	P02144	b4	461.1501	461.1519	0.0018
SEDEMK(+132.04)AS EDLK	1512.6478	505.2228	3	P02144	b2-H2O	199.0712	199.0719	0.0007
SEDEMK(+132.04)AS EDLK	1512.6478	505.2228	3	P02144	b3-H2O	314.0973	314.0988	0.0015
SEDEMK(+132.04)AS EDLK	1512.6478	505.2228	3	P02144	b4-H2O	443.1376	443.1414	0.0038
SEDEMK(+132.04)AS EDLK	1512.6478	505.2228	3	P02144	b7-NH3	906.343	906.3375	-0.0054
SEDEMK(+132.04)AS EDLK	1512.6478	505.2228	3	P02144	y1	147.1126	147.1128	0.0002
SEDEMK(+132.04)AS EDLK	1512.6478	505.2228	3	P02144	y2	260.1965	260.1968	0.0003
SEDEMK(+132.04)AS EDLK	1512.6478	505.2228	3	P02144	y3	375.2238	375.2238	0
SEDEMK(+132.04)AS EDLK	1512.6478	505.2228	3	P02144	y4	504.2673	504.2664	-0.0009
SEDEMK(+132.04)AS EDLK	1512.6478	505.2228	3	P02144	y5	591.2977	591.2984	0.0007
SEDEMK(+132.04)AS EDLK	1512.6478	505.2228	3	P02144	y6	662.3344	662.3355	0.0012
SEDEMK(+132.04)AS EDLK	1512.6478	505.2228	3	P02144	y10	1297.580 2	1297.5804	0.0002
SEDEMK(+132.04)AS EDLK	1512.6478	505.2228	3	P02144	y1-H2O	129.1021	129.1022	0.0001
SEDEMK(+132.04)AS EDLK	1512.6478	505.2228	3	P02144	y2-H2O	242.1859	242.1863	0.0004

SEDEMK(+132.04)AS EDLK	1512.6478	505.2228	3	P02144	y4-H2O	486.2564	486.2558	-0.0006
SEDEMK(+132.04)AS EDLK	1512.6478	505.2228	3	P02144	y6-H2O	644.3246	644.325	0.0003
SEDEMK(+132.04)AS EDLK	1512.6478	505.2228	3	P02144	y1-NH3	130.0861	130.0858	-0.0003
SEDEMK(+132.04)AS EDLK	1512.6478	505.2228	3	P02144	y2-NH3	243.1699	243.1698	0
SEDEMK(+132.04)AS EDLK	1512.6478	505.2228	3	P02144	y7[2+]	461.7388	461.7352	-0.0035
SEDEMK(+132.04)AS EDLK	1512.6478	505.2228	3	P02144	y9[2+]	591.774	591.7768	0.0027
DM(+15.99)ASNYK(+132.04)ELGFQG	1606.6798	804.3466	2	P02144	b2	263.0693	263.0702	0.0008
DM(+15.99)ASNYK(+132.04)ELGFQG	1606.6798	804.3466	2	P02144	b3	334.1061	334.1073	0.0012
DM(+15.99)ASNYK(+132.04)ELGFQG	1606.6798	804.3466	2	P02144	b2-NH3	246.0423	246.0432	0.0009
DM(+15.99)ASNYK(+132.04)ELGFQG	1606.6798	804.3466	2	P02144	y2	204.0976	204.0979	0.0003
DM(+15.99)ASNYK(+132.04)ELGFQG	1606.6798	804.3466	2	P02144	y3	351.1664	351.1663	-0.0001
DM(+15.99)ASNYK(+132.04)ELGFQG	1606.6798	804.3466	2	P02144	y2-H2O	186.0869	186.0873	0.0004
DM(+15.99)ASNYK(+132.04)ELGFQG	1606.6798	804.3466	2	P02144	y3-H2O	333.156	333.1557	-0.0003
DM(+15.99)ASNYK(+132.04)ELGFQG	1606.6798	804.3466	2	P02144	y2-NH3	187.0709	187.0709	0
DM(+15.99)ASNYK(+132.04)ELGFQG	1606.6798	804.3452	2	P02144	b2	263.0692	263.0702	0.001
DM(+15.99)ASNYK(+132.04)ELGFQG	1606.6798	804.3452	2	P02144	b3	334.1062	334.1073	0.0011
DM(+15.99)ASNYK(+132.04)ELGFQG	1606.6798	804.3452	2	P02144	b4	421.1388	421.1393	0.0005
DM(+15.99)ASNYK(+132.04)ELGFQG	1606.6798	804.3452	2	P02144	b6	698.2434	698.2455	0.0021
DM(+15.99)ASNYK(+132.04)ELGFQG	1606.6798	804.3452	2	P02144	b3-H2O	316.0957	316.0967	0.001
DM(+15.99)ASNYK(+132.04)ELGFQG	1606.6798	804.3452	2	P02144	b5-H2O	517.1679	517.1717	0.0038
DM(+15.99)ASNYK(+132.04)ELGFQG	1606.6798	804.3452	2	P02144	b2-NH3	246.0425	246.0432	0.0007
DM(+15.99)ASNYK(+132.04)ELGFQG	1606.6798	804.3452	2	P02144	y2	204.0975	204.0979	0.0004

DM(+15.99)ASNYK(+132.04)ELGFQG	1606.6798	804.3452	2	P02144	y3	351.1663	351.1663	0
DM(+15.99)ASNYK(+132.04)ELGFQG	1606.6798	804.3452	2	P02144	y4	408.1869	408.1877	0.0009
DM(+15.99)ASNYK(+132.04)ELGFQG	1606.6798	804.3452	2	P02144	y2-H2O	186.0869	186.0873	0.0004
DM(+15.99)ASNYK(+132.04)ELGFQG	1606.6798	804.3452	2	P02144	y3-H2O	333.1544	333.1557	0.0013
DM(+15.99)ASNYK(+132.04)ELGFQG	1606.6798	804.3452	2	P02144	y2-NH3	187.071	187.0709	-0.0001
DM(+15.99)ASNYK(+132.04)ELGFQG	1606.6798	804.3452	2	P02144	y3-NH3	334.1397	334.1393	-0.0004
